# Exploring Prajnamitra Maitreya Buddhists School Pekanbaru: Do leadership, work environment, and organisational culture influence the teachers’ competence and work performance?

**DOI:** 10.1371/journal.pone.0282941

**Published:** 2023-05-16

**Authors:** Fazal Mohamed Mohamed Sultan, Gunasegaran Karuppannan, Nyoto, Herni Lestari

**Affiliations:** 1 Faculty of Social Sciences and Humanities, Center for Research in Language and Linguistics, Universiti Kebangsaan Malaysia, Bangi, Malaysia; 2 Faculty of Education and Social Sciences, Open University Malaysia, Petaling Jaya, Malaysia; 3 Faculty of Business, Institut Bisnis Dan Teknologi Pelita, Pekanbaru, Indonesia; Universiti Pertahanan Nasional Malaysia, MALAYSIA

## Abstract

School administration must pay attention to professional teachers’ roles rather than nonprofessional teachers as part of important human resources in imparting learning. This study aims to analyse the influence of leadership, work environment, and organisational culture on the teachers’ competence and performance in Prajnamitra Maitreya Foundation Pekanbaru, Indonesia. A total of 57 teachers participated in this research. A descriptive analysis of questionnaires and hypothesis analysis using path analysis was used to analyse the data from the saturated sampling method, where 57 teachers became the sample and were categorised based on age, gender, level of education, years of service, and work unit. Using Smart PLS (Partial Least Squares), this research revealed that leadership and work environment positively but non-significant affect the teachers’ competence. Meanwhile, organisational culture has a positive and significant effect on the teachers’ competence but a non-significant positive effect on the teachers’ performance. Thus, the work environment and teacher’s competence have a positive and significant effect on the teacher’s performance, yet leadership has a negative and non-significant effect on the teacher’s performance.

## Introduction

An organisation will run well if workers cooperate reasonably with good human resources management. As the founder of motivation theories, Abraham H. Maslow triggered many other motivation theories that claimed that an organisation needs to pay extra attention to developing human resources for more remarkable performance. School organisation is unique in giving service to develop children’s potential. In this case, it also needs support from the Principal, teachers, staff, and Parent Teacher Associations [1]. To improve a school system is not only about changing the rules, but it requires the way of thought and interaction among people because the primary key lies in each individual’s relationship with the institutional structure, starting from the Principal, teachers, staff, and Parent Teacher Associations [2–6].

Teachers play an essential and crucial role in developing school students in education. Meanwhile, the other aspects, such as leaders, staff, stakeholders, parents, infrastructures, foundations, and the public, cannot be overlooked. Indonesia Law Number 14 of 2005 about teacher and lecturer part IV article 20 stated that teachers must plan, impart, assess and evaluate the learning to do their job professionally. Teachers’ performance in Preschool, Elementary, Junior, and Vocational High Schools in Prajnamitra Maitreya Foundation in Pekanbaru, Indonesia, is as follows:

Based on Table 1, all the teachers can prepare a lesson plan for each unit. According to the respondents’ interviews, lesson plans are taken from the internet, available online and widely used in the lesson plan format. We know that teachers do administration matters but must also deal with students’ problems daily. Also, evaluating the lesson plan in writing is always impossible because they must manage their full-time classes. Therefore, it is difficult for schools to improve their teachers’ performance.

**Table 1:** Teachers; performance achievement in preschool, elementary, junior, and vocational high school in Prajnamitra Maitreya Foundation, Pekanbaru.

Unit	Description	Academic Year 2015–2016	Academic Year 2016–2017	Academic Year 2017–2018	Academic Year 2018–2019	Academic Year 2019–2020
Preschool	Preparing a lesson plan	100%	100%	100%	100%	100%
	Implementing a lesson plan	57.14%	71.43%	85.71%	85.71%	81.82%
	Evaluating a lesson plan	57.14%	85.71%	85.71%	85.71%	90.91%
Elementary	Preparing a lesson plan	100%	100%	100%	100%	100%
	Implementing a lesson plan	73.08%	74.07%	71.43%	75.00%	78.57%
	Evaluating a lesson plan	76.92%	74.07%	78.57%	89.29%	89.29%
Junior High School	Preparing a lesson plan	100%	100%	100%	100%	100%
	Implementing a lesson plan	44.44%	47.06%	50.00%	65.22%	66.67%
	Evaluating a lesson plan	55.56%	64.71%	70.00%	60.87%	74.07%
Vocational High School	Preparing a lesson plan	100%	100%	100%	100%	100%
	Implementing a lesson plan	44.44%	47.06%	50.00%	65.22%	66.67%
	Evaluating a lesson plan	55.56%	64.71%	70.00%	60.87%	74.07%

Source: Metta Maitreya School, Pekanbaru, 2021.

In Table 1, there is always a target of 100% achievement at each stage, such as developing, implementing, and evaluating a lesson plan for each level, namely preschool, elementary, junior high, and vocational high school. However, no one level of education can fulfil the targets. Only the Preschool level has the highest achievement, with a rate of 90.91% at the evaluation level. The achievement targets in Table 1 could not be met for several reasons, which were ascertained by researchers conducting this study using observations and interviews. As shown in Table 1‘s explanations, several factors contribute to teachers’ inability to meet the targets, such as their excessive workload or the fact that they must perform administrative duties in addition to their teaching duties.

Aside from that, teachers are encouraged to be professional and competent, in accordance with the Law on the National Education System of the Republic of Indonesia 2005 number 14 [7], which contains four fundamental rights: pedagogic competence (teaching), personal competence, social competence, and competence acquired through professional education.

A highly competent teacher will do their best to conduct an innovative learning process [8,9]. The quality of the graduates reflects the teachers’ competence and quality in teaching. We will see the student’s final achievement in every unit of Prajnamitra Maitreya Foundation, Pekanbaru.

Table 2 shows a fluctuation in students’ final exam scores in every unit. This may indirectly reflect upon teachers’ professionalism and competency. Teachers’ ability to educate and produce high-quality graduates indicates competence. Effective learning comes from operative teaching [10]. Thus, teachers’ professionalism and qualifications must guarantee competent and brilliant students [11].

**Table 2:** Student’s final achievement in each unit of Prajnamitra Maitreya Foundation, Pekanbaru.

Unit	Academic Year 2015–2016	Academic Year 2016–2017	Academic Year 2017–2018	Academic Year 2018–2019	Academic Year 2019–2020
Preschool	Highly Developed	Highly Developed	Highly Developed	Highly Developed	Highly Developed
Elementary	78.1	77.4	69.7	69.6	77.4
Junior High School	82.74	81.10	87.84	75	90.83
Vocational High School	No Graduation		69.02	79.59	90.43

Source: Metta Maitreya School, Pekanbaru, 2021.

Research related to teachers’ competence and performance has been conducted in this last decade with mixed results. A teacher’s performance means a set of actions, attitudes, and behaviours in the teaching-learning environment that results in achieving educational goals for students. Soetopo [12] revealed that leadership and organisational culture influenced work performance. Teachers’ competence also affects their performance [13–15]. Pujiastuti & Rozi [16] also showed that teachers’ competence and the work environment influenced teachers’ performance. In contrast, some researchers revealed that teachers’ competence did not affect teachers’ performance [17–19]. Anggrayni et al. [20] proved that the work environment affected teachers’ performance, but another research showed no correlation between the work environment and teachers’ performance. Rahardjo [17] verified that leadership and the work environment influenced teachers’ performance. Otherwise, Sampurno & Wibowo [21] proved that the work environment did not directly affect teachers’ performance, but leadership impacts teachers’ performance [22]. Yani & Indrawati [23] revealed that the work environment influenced the teachers’ competence. There is a significant effect between leadership and organisational culture on teachers’ performance [24,25]. In addition, organisational culture also stimulates teachers’ performance [26–28]. Yusuf [29] disclosed that leadership and organisational culture harmed teachers’ performance. Putra et al. [26] proved no significant effect of leadership on teachers’ performance. While Indajang [30] also unveiled that organisational culture did not influence teachers’ performance. Another research showed that leadership and organisational culture positively influenced teachers’ competence [31]. Abidin et al. [32] unveiled that the work environment did not directly affect teachers’ competence.

Based on the phenomena above, the problem in this research is whether leadership, working environment, and organisational culture significantly affect teachers’ competence and performance and whether the teachers’ competence influences the teachers’ performance. Therefore, this research examines and analyses the effect of leadership, working environment, and organisational culture on teachers’ competence and work performance and the effect of teachers’ competence on the teachers’ performance in Prajanamitra Maitreya Foundation Pekanbaru, Indonesia. Hopefully, this research will contribute both, in theory, to the development of similar studies in the education field and practical development as feedback for improving the teachers’ competence and performance.

## Literature review and hypothesis

### Leadership

It can influence a person or an organisation to work willingly and effectively in achieving the organisation’s vision in certain circumstances [33,34]. Leadership in an organisation is crucial in affecting decision-making. Hence, it must be directed to management procedures regarding the organisation’s operational function [35]. Leadership’s biggest challenge is deciding wisely, which can influence people at every moment [36]. A leader can do the organisational activity by asking, motivating, influencing, and even forcing them to reach a goal. He also has excellent communication skills to make a great bond with the employee [37]. Successful leadership is affected by the working environment. Thus, it needs three prominent roles: leader, follower, and surrounding environment [1]. Leadership style is determined by the characteristics of the followers and the environment [38]. Communication skills, personality, intelligence, employee characteristics, and goal achievement are the factors that can affect leadership [4,39]. An effective leader is skilful in using the leadership style which builds upon the situation required. On the other hand, others might change their style, confusing them [40].

The leader also influences school development. The learning process defines the quality of education with the Principal’s capability to lead and manage professionally [41]. The Principal manages the school to achieve the best education quality [24,42,43]. The Principal’s leadership style needs to be modified, and the school organisation’s development needs to improve [44]. The Principal is the most critical component in developing the school, both in management and quality [45,46]. The dimensions regarding leadership are: (1) a well-defined strategy with excellent communication skills; (2) awareness of the employee and working environment; (3) ability to motivate; (4) maintaining teamwork; and (5) appreciating the differences and beliefs [47].

### Work environment

In management literature, very little attention is given to the impact of the work environment towards working creatively and innovatively. Aspects such as interior design, lighting, and circulation are necessary for building work motivation [48]. Knowledge and a modern perspective on the workplace must prepare the work environment to shape and confine employees’ behaviour [49]. Increasing productivity will focus on individual motivation and work environment infrastructure [50]. As the prominent factor stimulating work, the work environment will develop the employees’ economic growth [51]. A good work environment will support each individual to have a strong tenure, convince them to have healthy competition, and work effectively even under pressure [52]. The work environment holds the primary key in the individual variables [53].

An excellent work environment relates to teachers’ motivation to gain productivity in teaching and educating [16,21]. The work environment dimensions are divided into physical environments such as lighting, sound, aerial, infrastructure, security, and safety. In contrast, the non-physical environment is the relationship between the leader, teacher, and staff [54,55].

### Organisational culture

The study of organisational culture started in the early 1980s. It has adapted to an organisation’s new human values and eras [56]. Organisational culture begins from the habit, tradition, and common ways of doing things in an organisation based on past achievements [57]. Culture in an organisation is a beneficial tool for reaching the goal of organisation activity [58]. Organisational culture is a subject that failed to get specific attention from researchers [59]. It is the soul of an organisation because it consists of philosophy, vision and mission, values, and other things followed by the people [60]. It begins with forming the value and norms and passes on among individuals through social learning, role modelling, and observation. Ultimately, it helps the employee deal with external pressure threatening the organisation’s continuity and internal integration [61]. Organisational culture is a set of beliefs, values, norms, customs, and behaviours that influence how people think, feel, and act [62,63]. It is not defined but identifies how people work and behave to achieve their goals [40].

School culture is a group of norms, values, beliefs, rituals, ceremonies, symbols, and stories describing the school’s distinctive features [64]. It refers to every level of education. The vision and mission at school indicate the existence of culture because it proves that school and company cultures are equal [65]. School organisational culture gives the identity, achievement orientation, behaviour standard, and various ways of working and determines the future [66]. The dimensions in organisational culture are (1) Self-awareness to gain satisfaction in working, self-improvement, rule-obeying, and high service; (2) aggressiveness and determination in reaching the goal; (3) mutual respect, hospitality, openness, sensitivity to the needs of students; (4) Creative performance results in quantity and quality; (5) Team orientation with teamwork, communication, cooperation, involvement, commitment [47].

### Competence

Competence is the primary key to deciding an organisation’s effectiveness and success [62]. Competence means being qualified and capable of developing and well-functioning [67]. It can be related to the ability to problem-solving, cognitive skills, self-concept, behaviour, or value [68]. Competence can be the fundamental consideration in recruiting, selecting, planning, and evaluating work performance in developing human resources [69]. Competence differentiates one’s behaviour from the others [70]. It is crucial because the decision will be taken wrongly without the direction of competence [71].

In Indonesia Act, Number 14 of 2005 about Teachers and Lecturers, teachers as professional educators have the prominent roles of educating, teaching, guiding, training, assessing, and evaluating students from preschool, primary to high school. Teachers’ competence will influence teachers’ performance pedagogically, personally, and professionally [72]. A teacher must have academic, competent, physical, and mental conditions to reach the national education goal [73]. The dimension of teachers’ competence are: (1) Having knowledge and skill from the formal or informal institution; (2) being Skillful in teaching and solving a problem effectively; (3) Uphold the organisation’s ethics, acting positively and kindly; (4) Work properly [47].

### Work performance

It is the human work process [74]. Work performance also contributes to decision-making regarding salary based on achievement, promotion, and recruitment in building a positive team rapport [75,76]. Work performance is an organisation’s ability to manage human resources to reach its purpose [77,78]. Poor work performance will cause negative achievement and unpleasant results [79,80].

Teachers’ performance is the output from doing the task based on their ability, experience, sincerity, and time management [16,81]. National Education Ministerial Regulation Number 35 of 2010 mentioned that teachers’ performance assesses process and work achievement in doing their obligation. A professional teacher is knowledgeable in their teaching field, methods of teaching, and motivating students and comprehends social and human essence [16]. A teacher’s action is working with high morale, discipline, spirit, and dedication and living up to their profession [22]. The dimension of teachers’ performance are: (1) having a target in fulfilling their job, (2) The quality of achievement, (3) Punctuality in working, (4) Attitude regarding work quality to be reliable, accountable, open, and responsible [47,82].

### The effect of leadership on teachers’ competence

Leadership is defined as inspiring others to do their best to get what they want in developing and communicating vision, motivating employees, and keeping the commitment [40]. Soetopo [12] proved that leadership positively affects teachers’ competence. However, [26,31] revealed the result in contrast. Therefore, the first hypothesis is:

H1. Leadership has a significant effect on teachers’ competence in Prajnamitra Maitreya Foundation Pekanbaru.

### The effect of work environment on teachers’ competence

If the work environment is uncomfortable, it will disturb teachers’ competence. In contrast, a good work environment can create competence [23]. Furthermore, research showed that the work environment significantly affects teachers’ competence [16]. However, Abidin et al. [32] revealed that the work environment does not directly affect teachers’ competence. Therefore, the second hypothesis is:

H2. The work environment significantly affects teachers’ competence in Prajnamitra Maitreya Foundation Pekanbaru.

### The effect of organisational culture on teachers’ competence

Organisational culture helps shape the teachers’ behaviour in improving teachers’ competence to support teachers’ professionalism. The research revealed that organisational culture positively and significantly affects teachers’ competence [12,31]. On the contrary, [30] showed that it does not affect the teachers’ competence. Therefore, the third hypothesis is:

H3. Organisational culture significantly affects teachers’ competence in Prajnamitra Maitreya Foundation Pekanbaru.

### The effect of leadership on teachers’ work performance

Outstanding leadership can improve employees’ work performance to maximise output and reach the goal effectively and efficiently [60]. Teachers’ work performance can be achieved if the Principal does his or her task responsibly [25]. Transactional or transformational leadership can positively influence work performance [83]. The research revealed that leadership influenced work performance [2,14,17,21,30,83–86]. Nevertheless, [29] revealed that it does not affect the teachers’ performance. Therefore, the fourth hypothesis is:

H4. Leadership has a significant effect on teachers’ work performance in Prajnamitra Maitreya Foundation Pekanbaru.

### The effect of work environment on teachers’ work performance

The work environment helps improve teachers’ performance [87]. Creating a supportive environment of mutual respect and teamwork will significantly affect teachers’ performance [25]. A relaxing, harmonious, cosy, and comfortable environment at work is the main factor in building significant interaction in accomplishing work. Therefore, the work environment is the most dominant factor [16,17,88]. On the other hand, some research showed that the work environment had no significant correlation with teachers’ performance [18,21,49]. Thus, the fifth hypothesis is:

H5. The work environment has a significant effect on teachers’ work performance in Prajnamitra Maitreya Foundation Pekanbaru.

### The effect of organisational culture on teachers’ work performance

Organisational culture determines teachers’ performance which needs to be optimised at school [89]. It has a positive and significant effect on teachers’ performance [2,25,26,28,86] yet Yusuf [29] revealed the result vice versa. Hence, the sixth hypothesis is:

H6. Organisational culture has a significant effect on teachers’ work performance in Prajnamitra Maitreya Foundation Pekanbaru.

### The effect of teachers’ competence on teachers’ performance

There is a positive bond between professional competence and teachers’ performance. If competence improves, so does work performance [90]. A highly competent teacher will use his or her ability and capability to create an innovative learning process, including pedagogic, personality, professional, and social competence [9,15]. Excellent teachers’ competence will positively affect teachers’ performance [8,12,16,30,89]. In opposition, others showed that teachers’ competence did not affect teachers’ performance [17,18]. Thus, the seventh hypothesis is:

H7. Teachers’ competence has a significant effect on teachers’ work performance in Prajnamitra Maitreya Foundation Pekanbaru.

### Structural model path diagram

**Fig 1:**
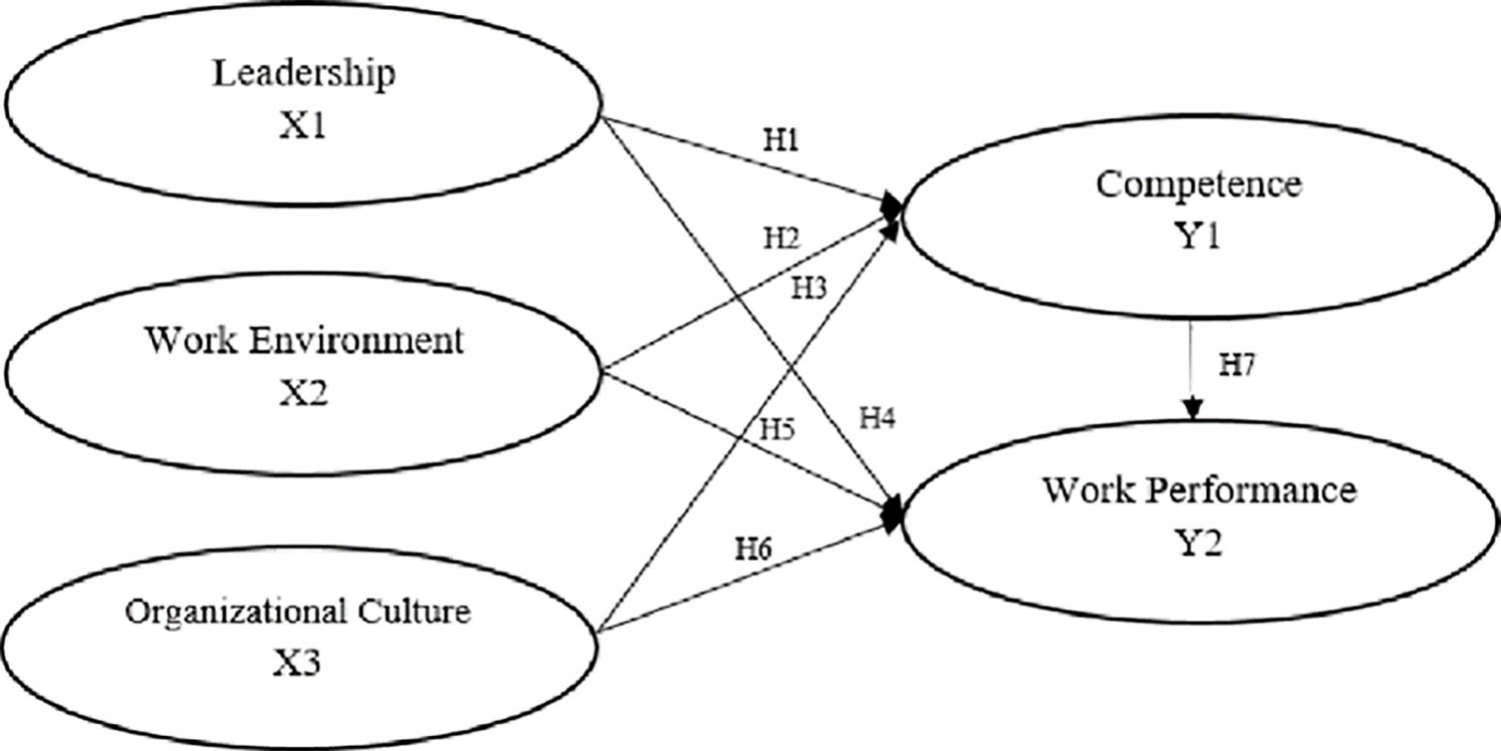
Structural model path diagram. Source: Processed Data, 2020.

The model measured in this research reflects the hypothesis to show the effect, as seen in Fig 1.

## Research methodology

### Research ethics statement

This research has obtained written approval from the leader of Prajnamitra Maitreya Foundation, Pekanbaru and the respondents. Prajnamitra Maitreya Foundation, Pekanbaru, Indonesia, granted permission for this research at Metta Maitreya School. The permission number is 055.1/L-YPM/XII/2020. All ethics procedures regarding informed consent, withdrawal, and anonymity assurance were followed at all research process stages. All the participants were emailed an information sheet with study details and a consent form. Written informed consent from all participants was obtained before they participated. All the staff agreed to participate and emailed a signed consent form indicating their participation.

### Research instrument

This is a survey research with a questionnaire in google forms due to the Covid-19 pandemic. All respondents gave their opinion in the answer blanks provided. The qualitative answer was converted to quantitative. Every answer is given a numeric score with a Likert Scale of 1 as “Strongly Disagree” to 5 as “Strongly Agree”.

### Population and sample

The population in this research are all teachers from Metta Maitreya School, the school directed by Prajnamitra Maitreya Foundation Pekanbaru, Indonesia. It consists of teachers from preschool, primary, and high school. A saturated sampling method was used in which the entire population of as many as 57 teachers became the sample and was categorised based on age, gender, level of education, years of service, and work unit.

### Place and time of research

The research was conducted in Prajnamitra Maitreya Foundation Pekanbaru directing Metta Maitreya School in Tuanku Tambusai Street, Puri Nangka Sari Complex, Marpoyan Damai District, Tangkerang Barat Sub-district, Pekanbaru City, Riau Province, Indonesia. The time of research starts from January 1^st^ until January 28^th^ 2021.

### Data collection technique and source

The primary data collection technique is from the questionnaires the 57 teachers have filled up. The PATH data analysis technique was used to conduct this research.

## Results

### General description of Prajnamitra Maitreya Foundation Pekanbaru

Prajnamitra Maitreya Foundation Pekanbaru was founded in 2004 by religious figures in Riau Province with a total of ±14.000m^2^ land and finally built Metta Maitreya School Pekanbaru starting from preschool, primary, secondary, and vocational high school. There are 84 employees, including 57 teachers and 27 staff.

### Descriptive analysis

#### Respondents’ characteristic analysis

This general description will inform the respondent’s characteristics Prajnamitra Maitreya Foundation Pekanbaru that is categorised based on age, gender, level of education, years of service, and work unit as follows:

Table 3 shows that teachers in Prajnamitra Maitreya Foundation Pekanbaru are dominated by the group of 21–30 years old, with 37 people or 64,91%. Sixteen people are from the group of 31–40 years old, or 28,07%. The age above 40 represents two people or 3,51%, and above 21 years old (19 years old for exact) represents two people or 3,51%. Most of the teachers are young, probably not having much experience in teaching. However, the researchers observed that the creativity in teaching, the willingness to improve themselves, and the ability to adapt to new technology are much better than others, which are indeed needed in this globalisation era.

**Table 3:** Respondents demographic profile.

Characteristic	Category	Frequency	%
Age	< 21 years old	2	3,51%
	21–30 years old	37	64,91%
	31–40 years old	16	28,07%
	> 40 years old	2	3,51%
Gender	Male	14	24,56%
	Female	43	75,44%
Level of Education	High School	7	12,28%
	Diploma	1	1,75%
	Undergraduate	48	84,21%
	Postgraduate	1	1,75%
Years of Service	1–5 years	44	77,19%
	6–10 years	10	17,54%
	> 10 years	3	5,26%
Work Unit	Preschool	8	14,04%
	Elementary	25	43,86%
	High School	24	42,11%

Source: Processed Data, 2020.

Regarding gender, it is dominated by female teachers, with 43 people or 75,44%. Millennial women around 22 to 38 are more involved in education [91]. However, the teachers’ recruitment at school is not merely based on gender but competence and performance.

From the level of education, they are primarily undergraduates, with 48 people or 84,21%. Seven people, or 12,28%, graduated from high school. 1,75% or one person did a diploma, and the other did a postgraduate diploma. According to Indonesian Law Number 14 of 2005 Article 9 and Government Regulation Number 74 of 2008, a teacher must be at least a diploma holder or a graduate. The need for high school graduate teachers is to teach a specific subject, namely the Chinese Language and Moral Ethics. These subjects’ teachers are still hard to recruit, but they are recruited because of their ability in these subjects. However, they are in the progress of studying the undergraduate program,

Regarding years of service, most teachers have worked for five years representing 77,20% or 44 people. Ten people have been working for almost ten years, and only three can reach more than ten years. Ningsih [92] stated that there is no difference between years of service and professionalism in working. It seems that it is not easy to find a loyal employee nowadays.

Most teachers in Prajnamitra Maitreya Foundation Pekanbaru work in the elementary unit, with 25 from 57. Besides, 24 work in high school and eight at preschool. This is merely based on the number of students in that unit. The more students, the more teachers are required.

### Data analysis technique

Path analysis is used for analysing with SPSS (Statistical Package for Social Science) Program Version 22 with the analysis statistic method, descriptive statistic, reliability analysis, and ANOVA. The validity test used Smart PLS 3.0.

### Questionnaire testing

*Validity and Reliability Test*. Minimum standard of validity with validity index score >0,3. Whilst the reliability test is *Cronbach’s Alpha* statistic test with a minimum score of 0,70 [93]. The result of the validity and reliability test is shown in Table 4.

**Table 4:** Validity and reliability test.

Variable	Indicator	R Count	R Table	Result	*Cronbach’s Alpha*	*Composite Reliability*	Result
**Leadership** **(X1)**	X.1	0.851	0.3	Valid	0,956	0.961	Reliable
	X.2	0.882					
	X.3	0.829					
	X.4	0.777					
	X.5	0.853					
	X.6	0.715					
	X.7	0.857					
	X.8	0.817					
	X.9	0.798					
	X.10	0.765					
	X.11	0.829					
	X.12	0.743					
	X.13	0.795					
**Work Environment** **(X2)**	X.1	0.851	0.3	Valid	0,876	0.899	Reliable
	X.2	0.882					
	X.3	0.829					
	X.4	0.777					
	X.5	0.853					
	X.6	0.715					
	X.7	0.857					
	X.8	0.817					
	X.9	0.799					
**Organisational Culture** **(X3)**	X.1	0.615	0.3	Valid	0,954	0.960	Reliable
	X.2	0.712					
	X.3	0.818					
	X.4	0.769					
	X.5	0.793					
	X.6	0.831					
	X.7	0.803					
	X.8	0.848					
	X.9	0.868					
	X.10	0.869					
	X.11	0.848					
	X.12	0.848					
	X.13	0.820					
**Competence** **(Y1)**	Y1.1	0.805	0.3	Valid	0,914	0.931	Reliable
	Y1.2	0.818					
	Y1.3	0.652					
	Y1.4	0.844					
	Y1.5	0.841					
	Y1.6	0.786					
	Y1.7	0.811					
	Y1.8	0.767					
**Work Performance** **(Y2)**	Y2.1	0.767	0.3	Valid	0,961	0.967	Reliable
	Y2.2	0.789					
	Y2.3	0.906					
	Y2.4	0.873					
	Y2.5	0.841					
	Y2.6	0.824					
	Y2.7	0.891					
	Y2.8	0.917					
	Y2.9	0.931					
	Y2.10	0.869					

Source: Processed Data by SPSS 22 and Smart PLS 3.0, 2021.

### Multicollinearity test

The multicollinearity test ensures no perfect correlation among independent variables by seeing the *Variance Inflating Factor* (VIF) from the regression result. It must be <10 to show no correlation. The result of the multicollinearity test is shown in Table 5.

**Table 5:** Multicollinearity test.

Independent Variable	Dependent Variable	VIF	Result
X1: Leadership	Y1 = Competence	2,166	No multicollinearity data
X2: Work Environment		1,493	
X3: Organisational Culture		2,283	
X1: Leadership	Y2 = Work Performance	2,198	
X2: Work Environment		1,511	
X3: Organisational Culture		6,788	
Y1: Competence		6,446	

Source: Processed Data-Smart PLS 3.0, 2021.

### Determination coefficient test (r^2^)

To know how much percentage of the indicators are influenced by the dependent variables while others are influenced by other factors not mentioned in the research.

From Table 6, the *R Square* for work performance is 0,792, which means the correlation between work performance with independent variables is strong. The *R Square Adjusted* is 0,776, representing 77,6% of the variation in teachers’ performance, which the variation of leadership, work environment, organisational culture, and competence can be explained. In comparison, the other 22,4% are affected by different factors not mentioned in this research, such as motivation, compensation, commitment, job satisfaction, organisation climate, etc. [16,21,23,26–29,83,85,86].

**Table 6:** Determination coefficient test (R^2^).

Variable	*R Square*	*R Square Adjusted*
Work Performance (Y2)	0.792	0.776
Competence (Y1)	0.845	0.836

Source: Processed Data-Smart PLS 3.0, 2021.

*R Square* for competence is 0,845, which means there is a strong correlation among independent variables. The *R Square Adjusted* is 0,836, which represents 83,6% of the competence that the variation of leadership can explain, work environment, and organisational culture, while the others 16,4%, influenced by others not mentioned in this research such as motivation, training, job satisfaction, etc. [12,17,18,23,31,32,89].

### Analysis of variance (ANOVA)

From the analysis of Anova, we can conclude that the average responses towards leadership are 4,386. It means the response is excellent. For example, the X1.13 indicator states, “Leader encourages the employee to have mutual respect in different beliefs”, with the highest average being 4,561 or excellent. The highest score is in the age of 21–30 years old, male, 1–5 years of service, with a diploma and postgraduate level of education in a preschool working unit.

This highest response is also shown in the responses based on gender, level of education, and work unit. The respondents have a great perception of their leaders in encouraging them to have religious tolerance. The one that needed to be focused on is the X1.12 indicator stating, “A leader respects all different opinions for a better purpose”, with the lowest score of 4,193 or good. The respondents’ characteristics are 21–30 years old, female, have more than ten years of service, and have a postgraduate level of education in the elementary working unit. This is also shown in respondents based on age, level of education, and years of service. This means that leaders need to pay more attention, listen more, and communicate better with their employees, enhancing interpersonal skills between the leader and employees.

The average responses towards the work environment are 4,626. It means the response is excellent. The X2.1 indicator states, “Lighting in the office will support work”, and the X2.6 indicator states, “Security in the office gives safety in working”, with the highest average of 4,754 or excellent. The highest score is below 21 and above 40 years old, female, with more than ten years of service, and with a diploma and postgraduate level of education in a preschool working unit. It means that lighting and security are crucial for effective working. The one that needed to be focused on is the X2.8 indicator stating. “Teachers have the same opportunity to improve themselves in working place”, with the lowest score of 4,368. The respondents’ characteristics are below 21 years old, male, more than ten years of service and postgraduate level of education in the elementary working unit. This is also shown in respondents based on age, gender, years of service, and work unit. This means that the respondents need to increase the opportunity to improve their performance.

The average of responses towards organisational culture is 4,614. It means the response is excellent. In the X3.4 indicator stating “Teachers give the best service for students”, the highest average is 4,614 or excellent. The highest score is between 31–40 years old, female, 6–10 years of service, with a diploma and postgraduate level of education in the elementary working unit. This is also shown in respondents based on age, gender, level of education, years of service, and work unit. It means that the teachers have tried their best to give students service since it is the main point in organisational culture. The one that needed to be focused on is the X3.5 indicator stating, “Teachers initiate in working and not always depend on the leaders’ instruction”, with the lowest score of 4,175 or good. The respondents’ characteristics are 31–40 years old, male, with 6–10 years of service, and undergraduate level of education in the elementary working unit. This is also shown in respondents based on age, years of service, and work unit. This means the respondents tend to depend on their leaders and do not initiate work because they worry they might be wrong.

The average responses towards competence are 4,409. It means the response is excellent. The Y1.7 indicator states, “Teachers have the hospitality in working”, with the highest average as 4,614 or excellent. The highest score is between 21–30 years old, female, 6–10 years of service, with a diploma and postgraduate level of education in a preschool working unit. This is also shown in respondents based on age, gender, level of education, years of service, and work unit. It means that the teachers have excellent hospitality and respect for one another. They tried their best to serve students since it is the main point in organisational culture. The one that needed to be focused on is the Y1.5 indicator stating, “Teachers can find the solution to every problem,” with the lowest score of 4,263. The respondents’ characteristics are 21–30 years old, male, with 6–10 years of service, and postgraduate level of education in the elementary working unit. This is also shown in respondents based on gender, level of education, and work unit. This means that the teachers still find solutions to problems with help from their leaders.

The average responses towards work performance are 4,596. It means the response is excellent. The Y2.10 indicator states, “Teachers have a responsibility in their job”, with the highest average as 4,614 or excellent. The highest score is below 20 years old, female, 6–10 years of service, with a diploma and postgraduate level of education in a preschool working unit. This is also shown in respondents based on age, gender, level of education, years of service, and work unit. It means that the teachers have a great responsibility in working. The one that needed to be focused on is the Y2.5 indicator stating, “Teachers, work is based on procedure to improve the quality of teaching”, with the lowest score of 4,386. The respondents’ characteristics are 21–30 years old, male, with more than ten years of service, and undergraduate level of education in the elementary working unit. This is also shown in respondents based on gender, years of service, level of education, and work unit. This means that the teachers need instructions in working based on procedures to improve the quality of teaching.

*Hypothesis Testing Path Analysis*. The hypothesis test result shows that the variables of leadership (X1) on competence (Y1), work environment (X2) on competence (Y1), leadership (X1) on work performance (Y2), and organisational culture (X3) on work performance (Y2) are not significant. On the other hand, organisational culture (X3) variables on competence (Y1) and work environment (X2) on work performance (Y2) have significant results. The result of these hypotheses can be seen in Table 7.

**Table 7:** Hypothesis testing result.

Variable	Original Sample (O)	Standard Deviation (STDEV)	T Statistics (|O/STDEV|)	P Values	Result
Leadership (X1) -> Competence (Y1)	0,071	0,097	0,734	0,463	Non-significant
Work Environment (X2) -> Competence (Y1)	0,053	0,055	0,967	0,334	Non-significant
Organizational Culture (X3) -> Competence (Y1)	0,836	0,072	**11,676**	**0,000** ***	Significant
Leadership (X1) -> Work Performance (Y2)	-0,042	0,116	0,365	0,715	Non-significant
Work Environment (X2) -> Work Performance (Y2)	0,131	0,079	**1,650**	**0,100** *	Significant
Organizational Culture (X3) -> Work Performance (Y2)	0,345	0,220	1,570	0,117	Non-significant
Competence (Y1) -> Work Performance (Y2)	0,514	0,193	**2,660**	**0,008** **	Significant

NB:

*Sig < 0.10

** < 0.05

***< 0,01.

Source: Processed Data Smart PLS 3.0, 2021.

The first hypothesis is that leadership has a positive and non-significant influence on teachers’ competence in Prajnamitra Maitreya Foundation Pekanbaru. The Original Sample (O) score is 0,071, which means a positive effect with a 0,463 p-value that means non-significant. Therefore, the first hypothesis is rejected. The second hypothesis is that the work environment has a positive and non-significant influence on teachers’ competence in Prajnamitra Maitreya Foundation Pekanbaru. The Original Sample (O) score is 0,053, which means a positive effect with a 0,334 p-value, meaning non-significant. Therefore, the second hypothesis is rejected. The third hypothesis is that organisational culture has a positive and significant influence on teachers’ competence in Prajnamitra Maitreya Foundation Pekanbaru. The Original Sample (O) score is 0,836, which is a positive effect with a 0,836 p-value that is significant. Therefore, the third hypothesis is accepted. The fourth hypothesis is that leadership has a negative and non-significant effect on teachers’ performance in Prajnamitra Maitreya Foundation Pekanbaru. The Original Sample (O) score is -0,042, which means a negative effect with a 0,715 p-value that means non-significant. Therefore, the fourth hypothesis is rejected. The fifth hypothesis is that the work environment positively and significantly affects teachers’ performance in Prajnamitra Maitreya Foundation Pekanbaru. The Original Sample (O) score is 0,131, which means a positive effect with a 0,100 p-value, meaning significance. Therefore, the fifth hypothesis is accepted.

**Fig 2:**
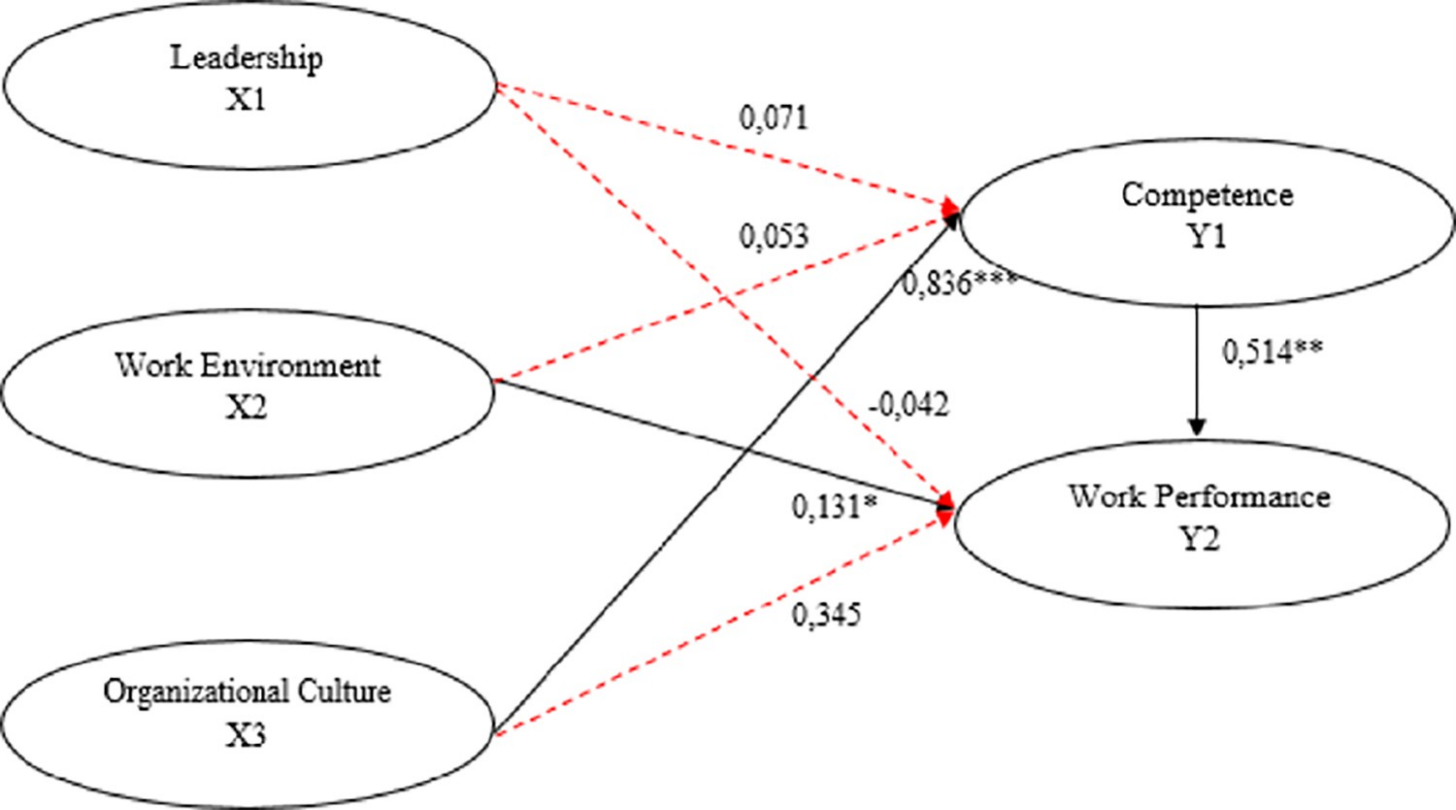
Path analysis result with SEM PLS. Source: Path Analysis, 2021.

The sixth hypothesis is that organisational culture has a positive and non-significant effect on teachers’ performance in Prajnamitra Maitreya Foundation Pekanbaru. The Original Sample (O) score is 0,345, which means a positive effect with a 0,117 p-value, meaning non-significant. Therefore, the sixth hypothesis is rejected. The seventh hypothesis is that teachers’ competence positively and significantly affects teachers’ performance in Prajnamitra Maitreya Foundation Pekanbaru. The Original Sample (O) score is 0,514, which means a positive effect with a 0,008 p-value which implies significance. Therefore, the seventh hypothesis is accepted. The path analysis result for these explanations can be seen in Fig 2.

### Structural equation analysis

From the structural equation, we can conclude that leadership positively affects teachers’ competence. If the leadership increases by 7,1% by assuming no intervention from other factors, the teachers’ competence can increase by 7,1%. The work environment has a positive effect on teachers’ competence. If the work environment increases by 5,3% by assuming no intervention from other factors, the teachers’ competence can increase by 5,3% as well. Organisational culture has a positive effect on teachers’ competence. If the organisational culture increases by 83,6% by assuming no intervention from other factors, the teachers’ competence can increase by 83,6%. Leadership harms teachers’ competence. If the leadership rises by 4,2% by assuming no intervention from other factors, the teachers’ performance can decrease by 4,2%. The work environment has a positive effect on teachers’ performance. If the work environment increases by 13,1% by assuming no intervention from other factors, the teachers’ performance can improve by 13,1%. Organisational culture has a positive effect on teachers’ performance. If the organisational culture increases by 34,5% by assuming no intervention from other factors, the teachers’ performance can improve by 34,5%. Teachers’ competence has a positive effect on teachers’ performance. If the teachers’ competence increases by 51,4% by assuming no intervention from other factors, the teachers’ performance can improve by 51,4%.

**Y _teachers’ competence_ = 0,071 Leadership + 0,053 Work Environment + 0,836 Organizational Culture**

**Y _teachers’ performance_ = (-0,042) Leadership + 0.131 Work Environment + 0,345 Organizational Culture + 0,514 Competence**

## Discussion

Leadership has a positive effect on teachers’ competence, which means that the improvement of leadership will subtly increase the teachers’ competence and affect only a few. Therefore, it is non-significant. It is supported by the research of Putra, et al. [26] and Syakir & Pardjono [31] and, on the contrary, by the study of Soetopo [12]. Leadership and competence are essential in determining an organisation’s effectiveness and success [62]. Leaders must motivate teachers to work significantly based on their competence resulting in qualified graduates [46]. Teachers in this foundation think that the leaders can encourage them, and mutual respect is present to learn from the leaders. Arman et al. [74] explained that teachers must be inclusive and objective without discrimination. He must communicate effectively, have empathy, and respect others. He will participate actively and supportively in the organisation if it meets their personal needs [71]. Teachers, too, feel that there is a need for a leader to listen to their opinion to help to develop the school.

The work environment positively affects teachers’ competence, which means that improving the work environment will also trivially increase their competence and affect only a few. Therefore, it is non-significant. It is supported by Abidin et al. [32] and in contrast with Soetopo’s [12] research. On the other hand, the teachers feel that the opportunity to promote themselves at school is lacking. The government and schools should find a system to help the teachers be promoted faster to keep their motivation high. They should provide an objective approach in their promotion system, not a subjective one. It also affects the competence level of the teachers as well.

Organisational culture positively affects teachers’ competence, which means that organisational culture improvement will also increase teachers’ competence with a considerable contribution. Therefore, it is significant. It is supported by the research of Soetopo [12] and Syakir & Pardjono [31] and in contrast with the study of Indajang [30]. Each organisation has an important culture that can affect the behaviour and attitude of the employees [62]. Without a solid culture, no organisation can accomplish its goal and survive in the competitive era [56]. Organisational culture is the soul of an organisation, with its philosophy, vision, and mission held by its people [60]. The organisational culture in this foundation is influential in the universal family concept that also influences how teachers think and act. Their acceptance towards this culture can be seen through the responses in this section. However, as observed in their questionnaire responses, these teachers still lack problem-solving competence.

Leadership hurts teachers’ performance means that the improvement of leadership will decrease the teachers’ performance in minor ways and affect only a few of them. Therefore, it is non-significant. It is supported by the research of Yusuf [29] and in contrast with the study of [17,21,24,25,30,83,84,86]. The leaders’ competence determines teachers’ outstanding performance [74]. Qualified leadership leads to skilled performance, knowledge, competence, motivation and job satisfaction [94]. From our analysis, good leadership does not always influence the teachers’ performance. The different circumstances in every research also contribute to different results compared to the theory in common. The questionnaire can also be biased since leadership itself has many dimensions. Since leadership only has one factor in improving performance, we must not overlook other factors such as achievement, learning preparation, commitment, and supervision [95]. It can also be because the leaders are probably not fully helpful in developing the teachers as they think of it as the foundation’s or the government’s job [94]. Therefore, the school must improve the leadership strategy to improve the teachers’ performance.

The work environment positively affects teachers’ performance, meaning that improving the work environment will also increase the teachers’ performance and significantly impact most teachers. Therefore, it is significant. It is supported by the research of [16,17] and in contrast with the study of [18,21,49]. The work environment will be conducive to increase work performance, leading to great productivity [50,52]. A good work environment will improve their ability to do their task [16,21,87]. Teachers feel that lighting and security will also help them to increase their work performance. A conducive work environment such as ample computers, ergonomic chairs, free stationeries and supportive senior colleagues will improve their work performance.

Organisational culture positively affects teachers’ performance, which means that organisational culture improvement will also increase the teachers’ performance with a small contribution by most teachers. Therefore, it is significant. It is supported by the research of Yusuf [29] and the research of [24,26–28,85,86]. Furthermore, teachers strongly agree about the importance of being responsible and giving excellent service to students. This means a tremendous organisational culture will aim for outstanding work performance, indirectly improving teaching. The students will get the most from their teachers during every lesson.

Teachers’ competence positively affects teachers’ performance, which means that the improvement of organisational culture will also increase the teachers’ performance with a significant impact on most of the teachers. Therefore, it is significant. It is supported by the research of [12,16,30,89] and the research of Rahardjo [17] dan Sari [18]. Excellent teachers’ competence will improve teachers’ performance because professional teachers will produce qualified education [63]. Competence is a personal factor that can influence teachers’ performance [96]. Mastering their subject will become a solid reason for the teachers’ ability to do their tasks and be responsible as educators [74]. Knowing the competence level is required to know the level of performance [69]. The competence standard is an approach to control the employee’s performance by learning management [97]. National Education Ministry Regulation Number 35 of 2010 stated that the teachers’ performance could be improved if they reach the right competence. If they can find solutions for the mentioned problem, then they can provide excellent work performance with their skills.

## Conclusion

It can be concluded that leadership positively affects teachers’ competence in Prajnamitra Maitreya Foundation Pekanbaru, which means that the improvement of leadership will also subtly increase the teachers’ competence and affect only a few of them. The work environment positively affects teachers’ competence in Prajnamitra Maitreya Foundation Pekanbaru, which means that improving the work environment will also increase the teachers’ competence and affect only a few. Then, organisational culture positively affects teachers’ competence in Prajnamitra Maitreya Foundation Pekanbaru. This means that organisational culture improvement will also increase the teachers’ competence with a massive contribution for most teachers. However, leadership negatively affects teachers’ performance in Prajnamitra Maitreya Foundation Pekanbaru, which means that leadership improvement will decrease the teachers’ performance in a minor way and affect only a few of them. The work environment positively affects teachers’ performance in Prajnamitra Maitreya Foundation Pekanbaru, which means that improving the work environment will increase the teachers’ performance and significantly impact most teachers. Organisational culture positively affects teachers’ performance in Prajnamitra Maitreya Foundation Pekanbaru, which means that organisational culture improvement will also increase the teachers’ performance, however, with a small contribution. Lastly, teachers’ competence positively affects teachers’ performance in Prajnamitra Maitreya Foundation Pekanbaru, which means that organisational culture improvement will significantly increase teachers’ performance, impacting most teachers.

The implication of this research is four suggestions that the foundation can utilise. First, there is a necessity to improve the teachers’ competence and performance to produce qualified graduates through motivation, improving the work environment, and training. Second, the foundation should consider all employees’ opportunities to develop their abilities. Third, a strict recruitment process is also needed. Finally, the leaders’ roles are crucial in dealing with the employee, so paying attention to the leader is necessary.

The implication of these teachers are suggestions that the teachers can implement. First, teachers must not overlook the importance of teachers’ competence, so they must keep improving themselves in many ways, such as self-learning or training. Second, teachers must initiate wisely in working to solve problems quickly. Third, if there is an issue in solving problems, they must discuss it with their colleagues or leaders. Fourth, there are gaps in this research that other researchers can improve. The researchers can differ the variables to get various results. Researchers also can find more research gaps concerning these variables. Fourth, the research can be done in multiple foundations or schools.

## Supporting information

S1 File (DOCX)

S2 File (ZIP)
