# Supplementary material for: Exploring Prajnamitra Maitreya Buddhists School Pekanbaru: Do leadership, work environment, and organisational culture influence the teachers’ competence and work performance?
Source: PLoS One. 2023 May 16;18(5):e0282941. doi: 10.1371/journal.pone.0282941 (PMC10187918; doi:10.1371/journal.pone.0282941)
Supplement: S1 File — (DOCX) [file pone.0282941.s001.docx]

**ANGKET PENELITIAN**

**(Diisi oleh Guru)**

**Identitas Responden:**

1. Nama : __________________________________________________

2. Tempat Kerja : __________________________________________________

3. Jabatan/ Pangkat : __________________________________________________

4. Pendidikan Terakhir : _______ 1. SMA/ SMK 2. D3 3. S1 4. S2

5. Masa Kerja : ____________ tahun

6. Usia Saat Ini : ____________ tahun

7. Jenis Kelamin : _______ 1. Laki-laki 2. Perempuan

**Petunjuk Pengisian:**

Bapak/ Ibu diminta untuk memberikan tanda “**√**” pada pilihan yang sesuai.

**Keterangan:**

| **Tingkat Kepuasan** |
| --- |
| 1. STS = Sangat Tidak Setuju |
| 2. TS = Tidak Setuju |
| 3. CS = Cukup Setuju |
| 4. S = Setuju |
| 5. SS = Sangat Setuju |

**Variabel Kepemimpinan**

| **No** | **Deskripsi** | **STS** | **SS** | **KS** | **S** | **SS** |
| --- | --- | --- | --- | --- | --- | --- |
| 1 | Pimpinan memiliki strategi yang realistis |  |  |  |  |  |
| 2 | Strategi disampaikan dengan jelas sehingga dapat diterima bawahan |  |  |  |  |  |
| 3 | Tujuan yang ditetapkan pimpinan harus dapat membawa perubahan yang lebih baik |  |  |  |  |  |
| 4 | Pimpinan memberikan perhatian secara personal untuk memotivasi kerja bawahan |  |  |  |  |  |
| 5 | Pimpinan peduli terhadap setiap masalah yang terjadi pada bawahan |  |  |  |  |  |
| 6 | Pimpinan memperhatikan kenyamanan lingkungan kerja bawahan di dalam bekerja |  |  |  |  |  |
| 7 | Pimpinan memotivasi bawahan untuk membekali diri dengan berbagai keahlian dalam usaha meningkatkan kompetensi |  |  |  |  |  |
| 8 | Pimpinan memotivasi bawahan untuk memiliki tekad menyelesaikan tugas hingga tuntas |  |  |  |  |  |
| 9 | Pimpinan mengajak bawahan untuk berorientasi pada kualitas |  |  |  |  |  |
| 10 | Pimpinan mengajak bawahan untuk bekerja harmonis dalam tim |  |  |  |  |  |
| 11 | Pimpinan dapat menyelesaikan setiap konflik dengan baik |  |  |  |  |  |
| 12 | Pimpinan menghargai setiap perbedaan pendapat untuk tujuan ke arah yang lebih baik |  |  |  |  |  |
| 13 | Pimpinan mengajak seluruh bawahan untuk menghormati perbedaan keyakinan |  |  |  |  |  |

**Variabel Lingkungan Kerja**

| **No** | **Deskripsi** | **STS** | **SS** | **KS** | **S** | **SS** |
| --- | --- | --- | --- | --- | --- | --- |
| 1 | Penerangan di ruang kerja yang baik mendukung pekerjaan. |  |  |  |  |  |
| 2 | Sirkulasi udara di ruang kerja yang baik mendukung pekerjaan. |  |  |  |  |  |
| 3 | Guru dapat bekerja nyaman jika tempat kerja tidak terganggu suara bising. |  |  |  |  |  |
| 4 | Penataan dekorasi di tempat kerja berpengaruh terhadap perasaan |  |  |  |  |  |
| 5 | Ruangan tidak pengab akan mendukung pekerjaan. |  |  |  |  |  |
| 6 | Keamanan di tempat kerja memberikan rasa aman dalam bekerja. |  |  |  |  |  |
| 7 | Hubungan harmonis antara pimpinan dan rekan kerja dapat mendukung pekerjaan. |  |  |  |  |  |
| 8 | Guru mendapatkan peluang untuk meningkatkan prestasi di tempat kerja. |  |  |  |  |  |
| 9 | Guru merasa aman bekerja tanpa khawatir kehilangan barang. |  |  |  |  |  |

**Variabel Budaya Organisasi**

| **No** | **Deskripsi** | **STS** | **SS** | **KS** | **S** | **SS** |
| --- | --- | --- | --- | --- | --- | --- |
| 1 | Guru mendapat kepuasan atas pekerjaan yang dilakukan |  |  |  |  |  |
| 2 | Guru berusaha mengembangkan kemampuan dirinya |  |  |  |  |  |
| 3 | Guru menaati segala peraturan yang ada |  |  |  |  |  |
| 4 | Guru berusaha memberikan pelayanan terbaik pada peserta didik |  |  |  |  |  |
| 5 | Guru berinisiatif dalam bekerja sehingga tidak selalu bergantung pada petunjuk pimpinan |  |  |  |  |  |
| 6 | Guru merencanakan pekerjaan dan menyelesaikan dengan baik |  |  |  |  |  |
| 7 | Rekan kerja saling menghargai, menghormati, dan melayani |  |  |  |  |  |
| 8 | Guru membantu rekan kerja yang kurang cakap dalam bekerja |  |  |  |  |  |
| 9 | Guru mengutamakan kualitas dalam menyelesaikan pekerjaan |  |  |  |  |  |
| 10 | Guru berusaha berinovasi menemukan hal baru dan berguna untuk perkembangan sekolah |  |  |  |  |  |
| 11 | Guru bekerja dengan efektif dan efisien |  |  |  |  |  |
| 12 | Tim kerja selalu berdiskusi dalam mengerjakan tugas agar dapat disinergikan |  |  |  |  |  |
| 13 | Masalah dalam tim selalu diselesaikan dengan baik |  |  |  |  |  |

**Variabel Kompetensi Guru**

| **No** | **Deskripsi** | **STS** | **SS** | **KS** | **S** | **SS** |
| --- | --- | --- | --- | --- | --- | --- |
| 1 | Guru memiliki pengetahuan yang cukup mendukung dalam pekerjaan yang ditangani |  |  |  |  |  |
| 2 | Guru berusaha belajar meningkatkan pengetahuan yang dimiliki |  |  |  |  |  |
| 3 | Keahlian teknis yang dimiliki guru sesuai bidang yang diajarkan |  |  |  |  |  |
| 4 | Guru mampu mengidentifikasi masalah yang timbul dalam pekerjaan yang ditangani |  |  |  |  |  |
| 5 | Guru mampu mencari solusi atas masalah yang timbul dalam pekerjaan yang ditangani |  |  |  |  |  |
| 6 | Guru berinisiatif dalam membantu rekan kerja menyelesaikan pekerjaan |  |  |  |  |  |
| 7 | Guru ramah dalam menghadapi sesama |  |  |  |  |  |
| 8 | Guru serius dalam menanggapi setiap keluhan yang muncul di sekolah |  |  |  |  |  |

**ANGKET PENELITIAN**

**(Diisi oleh Kepala Sekolah)**

**Identitas Guru yang Dinilai**

1. Nama : __________________________________________________

2. Tempat Kerja : __________________________________________________

Petunjuk Pengisian:

Bapak/ Ibu diminta untuk memberikan tanda “**√**” pada pilihan yang sesuai.

Keterangan:

| **Tingkat Kepuasan** |
| --- |
| 1. STS = Sangat Tidak Setuju |
| 2. TS = Tidak Setuju |
| 3. CS = Cukup Setuju\ |
| 4. S = Setuju |
| 5. SS = Sangat Setuju |

| **No** | **Deskripsi** | **STS** | **SS** | **KS** | **S** | **SS** |
| --- | --- | --- | --- | --- | --- | --- |
| 1 | Guru bekerja berpedoman pada target yang harus dipenuhi |  |  |  |  |  |
| 2 | Guru menjadikan target sebagai tantangan bukan rintangan untuk menjadi pribadi yang lebih baik |  |  |  |  |  |
| 3 | Kualitas kerja yang dihasilkan guru sesuai dengan standar yang ditetapkan |  |  |  |  |  |
| 4 | Guru komitmen dalam menjaga kualitas kerja |  |  |  |  |  |
| 5 | Guru bekerja berdasarkan prosedur yang ada untuk memenuhi kualitas |  |  |  |  |  |
| 6 | Guru memiliki keterampilan teknis untuk menyelesaikan tugas tepat waktu |  |  |  |  |  |
| 7 | Guru komitmen dalam menyelesaikan tugas tepat waktu |  |  |  |  |  |
| 8 | Guru melakukan pekerjaan dengan cara yang benar |  |  |  |  |  |
| 9 | Guru menaati perintah pimpinan dalam menyelesaikan tugas |  |  |  |  |  |
| 10 | Proses pekerjaan dilakukan dengan transparan sehingga dapat dipertanggungjawabkan |  |  |  |  |  |

**QUESTIONNAIRE**

**(For Teacher)**

**Respondent Identity:**

1. Name : __________________________________________________

2. Workplace : __________________________________________________

3. Position : __________________________________________________

4. Education : 1. High School 2. Diploma 3. Undergraduate 4. Postgraduate

5. Years of Service : ____________ years

6. Current Age : ____________ years old

7. Gender : 1. Male 2. Female

**Instruction:**

Please tick (**√**) based on your opinion

**Description:**

| **Level of Satisfaction** |
| --- |
| 1. SD = Strongly Disagree |
| 2. D = Disagree |
| 3. N = Neutral |
| 4. A = Agree |
| 5. SA = Strongly Agree |

**Leadership Variable**

| **No** | **Description** | **SD** | **D** | **N** | **A** | **SA** |
| --- | --- | --- | --- | --- | --- | --- |
| 1 | Leader has realistic strategies |  |  |  |  |  |
| 2 | The strategy is clearly conveyed so that it can be accepted by subordinates |  |  |  |  |  |
| 3 | The goals set by the leader must be able to bring about change for the better |  |  |  |  |  |
| 4 | Leader pays personal attention to motivate subordinates' work |  |  |  |  |  |
| 5 | Leader cares about any problems that occur to subordinates |  |  |  |  |  |
| 6 | Leader pays attention to the comfort of the work environment of subordinates |  |  |  |  |  |
| 7 | Leader motivates subordinates to equip themselves with various skills to improve competence |  |  |  |  |  |
| 8 | Leader encourages subordinates to have the determination to complete the task |  |  |  |  |  |
| 9 | Leader enhances subordinates to be quality oriented |  |  |  |  |  |
| 10 | Leader persuades subordinates to work harmoniously in a team |  |  |  |  |  |
| 11 | Leader can resolve every conflict well |  |  |  |  |  |
| 12 | Leader respects every difference of opinion for a better goal |  |  |  |  |  |
| 13 | Leader invites all subordinates to respect differences in beliefs |  |  |  |  |  |

**Work Environment Variable**

| **No** | **Description** | **SD** | **D** | **N** | **A** | **SA** |
| --- | --- | --- | --- | --- | --- | --- |
| 1 | Good lighting in the workspace supports the work |  |  |  |  |  |
| 2 | Good air circulation in the workspace supports the work |  |  |  |  |  |
| 3 | Teachers can work comfortably if the workplace is not disturbed by noise |  |  |  |  |  |
| 4 | The decorations in the workplace affects emotions |  |  |  |  |  |
| 5 | A not-stuffy room will support work |  |  |  |  |  |
| 6 | Safety in the workplace provides a sense of security at work |  |  |  |  |  |
| 7 | Harmonious relationships between leaders and co-workers can support work |  |  |  |  |  |
| 8 | Teachers get opportunities to improve performance in the workplace |  |  |  |  |  |
| 9 | Teachers feel safe working without worrying about losing things |  |  |  |  |  |

**Organizational Culture Variable**

| **No** | **Description** | **SD** | **D** | **N** | **A** | **SA** |
| --- | --- | --- | --- | --- | --- | --- |
| 1 | Teachers satisfy with their own work |  |  |  |  |  |
| 2 | Teachers try to develop their abilities |  |  |  |  |  |
| 3 | Teachers obey all rules at work |  |  |  |  |  |
| 4 | Teachers try to provide the excellent service to students |  |  |  |  |  |
| 5 | Teachers initiate in working so they do not always depend on the leader's instructions |  |  |  |  |  |
| 6 | Teachers plan the work and complete it well |  |  |  |  |  |
| 7 | Colleagues respect, honor, and care each other |  |  |  |  |  |
| 8 | Teachers help coworkers who are less proficient at work |  |  |  |  |  |
| 9 | Teachers prioritize quality in completing work |  |  |  |  |  |
| 10 | Teachers are being innovative for school development |  |  |  |  |  |
| 11 | Teachers work effectively and efficiently |  |  |  |  |  |
| 12 | The teamwork always discuss in doing task so that it can be synergized |  |  |  |  |  |
| 13 | Problems in the team are always resolved well |  |  |  |  |  |

**Teachers’ Competence Variable**

| **No** | **Description** | **SD** | **D** | **N** | **A** | **SA** |
| --- | --- | --- | --- | --- | --- | --- |
| 1 | Teachers have sufficient knowledge to support the work at hand |  |  |  |  |  |
| 2 | Teachers try to improve their knowledge |  |  |  |  |  |
| 3 | The teachers’ technical skills are matched to their own expertise |  |  |  |  |  |
| 4 | Teachers are able to identify problems that arise at work |  |  |  |  |  |
| 5 | Teachers are able to find solutions to problems that arise at work |  |  |  |  |  |
| 6 | Teachers initiate in helping colleagues completing work |  |  |  |  |  |
| 7 | Teachers are hospitable when dealing with others |  |  |  |  |  |
| 8 | Teachers are whole-hearted in responding to any complaints that arise at school |  |  |  |  |  |

**QUESTIONNAIRE**

**(For Principal/Headmaster)**

**Teacher’s Identity (that being assessed)**

1. Name : __________________________________________________

2. Workplace : __________________________________________________

**Instruction:**

Please tick (**√**) based on your opinion

**Description:**

| **Level of Satisfaction** |
| --- |
| 1. SD = Strongly Disagree |
| 2. D = Disagree |
| 3. N = Neutral |
| 4. A = Agree |
| 5. SA = Strongly Agree |

| **No** | **Description** | **SD** | **D** | **N** | **A** | **SA** |
| --- | --- | --- | --- | --- | --- | --- |
| 1 | Teacher works based on the targets given |  |  |  |  |  |
| 2 | Teacher sees targets not as obstacles but challenges to become a successful person |  |  |  |  |  |
| 3 | The quality of work produced by teacher is in accordance with the standards |  |  |  |  |  |
| 4 | Teacher is committed to maintain the quality of work |  |  |  |  |  |
| 5 | Teacher works based on standard operational procedures to meet the quality |  |  |  |  |  |
| 6 | Teacher has technical skills to accomplish work in time |  |  |  |  |  |
| 7 | Teacher is committed to complete tasks on time  Guru komitmen dalam menyelesaikan tugas tepat waktu |  |  |  |  |  |
| 8 | Teacher does the job in the right way |  |  |  |  |  |
| 9 | Teacher obeys the leader's instructions in completing the task |  |  |  |  |  |
| 10 | The work process is carried out transparently so that it can be accounted for |  |  |  |  |  |
