# Supplementary material for: Exploring Prajnamitra Maitreya Buddhists School Pekanbaru: Do leadership, work environment, and organisational culture influence the teachers’ competence and work performance?
Source: PLoS One. 2023 May 16;18(5):e0282941. doi: 10.1371/journal.pone.0282941 (PMC10187918; doi:10.1371/journal.pone.0282941)
Supplement: S2 File — (ZIP) [file pone.0282941.s002.zip › tabel uda olah anova.docx]

| Usia | <21 tahun | 21-30 tahun | 31-40 tahun | 41-50 tahun | Rata-rata | F-test | Sign |
| --- | --- | --- | --- | --- | --- | --- | --- |
| X1.1 | 5 | 4.432432 | 4.3125 | 4.5 | 4.561233 | 0.672 | 0.573 |
| X1.2 | 5 | 4.27027 | 4.375 | 4.5 | 4.536318 | 0.718 | 0.546 |
| X1.3 | 4.5 | 4.486486 | 4.4375 | 4.5 | 4.480997 | 0.029 | 0.993 |
| X1.4 | 4.5 | 4.351351 | 4.3125 | 4.5 | 4.415963 | 0.074 | 0.974 |
| X1.5 | 4.5 | 4.27027 | 4.5 | 4.5 | 4.442568 | 0.498 | 0.685 |
| X1.6 | 4.5 | 4.405405 | 4.375 | 4.5 | 4.445101 | 0.057 | 0.982 |
| X1.7 | 4.5 | 4.351351 | 4.25 | 4.5 | 4.400338 | 0.184 | 0.906 |
| X1.8 | 5 | 4.486486 | 4.375 | 4.5 | 4.590372 | 0.818 | 0.49 |
| X1.9 | 5 | 4.324324 | 4.4375 | 4.5 | 4.565456 | 1.17 | 0.33 |
| X1.10 | 5 | 4.486486 | 4.5625 | 4.5 | 4.637247 | 0.597 | 0.62 |
| X1.11 | 4.5 | 4.162162 | 4.25 | 4.5 | 4.353041 | 0.315 | 0.814 |
| X1.12 | 4.5 | 4.162162 | 4.1875 | 4.5 | 4.337416 | 0.298 | 0.827 |
| X1.13 | 5 | 4.594595 | 4.4375 | 4.5 | 4.633024 | 0.781 | 0.51 |

| Usia | <21 tahun | 21-30 tahun | 31-40 tahun | 41-50 tahun | Rata-rata | F-test | Sign |
| --- | --- | --- | --- | --- | --- | --- | --- |
| X2.1 | 5 | 4.702703 | 4.8125 | 5 | 4.878801 | 0.685 | 0.565 |
| X2.2 | 5 | 4.594595 | 4.875 | 5 | 4.867399 | 2.088 | 0.113 |
| X2.3 | 5 | 4.324324 | 4.75 | 5 | 4.768581 | 3.24 | 0.029 |
| X2.4 | 5 | 4.297297 | 4.8125 | 5 | 4.777449 | 3.573 | 0.02 |
| X2.5 | 5 | 4.675676 | 4.8125 | 5 | 4.872044 | 0.714 | 0.548 |
| X2.6 | 5 | 4.702703 | 4.8125 | 5 | 4.878801 | 0.572 | 0.636 |
| X2.7 | 5 | 4.648649 | 4.875 | 5 | 4.880912 | 1.262 | 0.297 |
| X2.8 | 4 | 4.459459 | 4.1875 | 4.5 | 4.28674 | 0.768 | 0.517 |
| X2.9 | 4 | 4.567568 | 4.6875 | 5 | 4.563767 | 1.284 | 0.289 |

| Usia | <21 tahun | 21-30 tahun | 31-40 tahun | 41-50 tahun | Rata-rata | F-test | Sign |
| --- | --- | --- | --- | --- | --- | --- | --- |
| X3.1 | 4.5 | 4.297297 | 4.3125 | 4.5 | 4.402449 | 0.118 | 0.949 |
| X3.2 | 4.5 | 4.486486 | 4.4375 | 4.5 | 4.480997 | 0.029 | 0.993 |
| X3.3 | 4.5 | 4.405405 | 4.375 | 4.5 | 4.445101 | 0.05 | 0.985 |
| X3.4 | 4.5 | 4.567568 | 4.75 | 4.5 | 4.579392 | 0.442 | 0.724 |
| X3.5 | 4.5 | 4.189189 | 4.0625 | 4.5 | 4.312922 | 0.436 | 0.728 |
| X3.6 | 4.5 | 4.459459 | 4.5 | 4.5 | 4.489865 | 0.021 | 0.996 |
| X3.7 | 4.5 | 4.459459 | 4.5 | 4.5 | 4.489865 | 0.019 | 0.996 |
| X3.8 | 4.5 | 4.324324 | 4.5625 | 4.5 | 4.471706 | 0.566 | 0.64 |
| X3.9 | 4.5 | 4.432432 | 4.375 | 4.5 | 4.451858 | 0.063 | 0.979 |
| X3.10 | 4.5 | 4.324324 | 4.4375 | 4.5 | 4.440456 | 0.202 | 0.895 |
| X3.11 | 4.5 | 4.405405 | 4.5 | 4.5 | 4.476351 | 0.115 | 0.951 |
| X3.12 | 4.5 | 4.324324 | 4.3125 | 4.5 | 4.409206 | 0.081 | 0.97 |
| X3.13 | 4 | 4.405405 | 4.5 | 4.5 | 4.351351 | 0.359 | 0.783 |

| Usia | <21 tahun | 21-30 tahun | 31-40 tahun | 41-50 tahun | Rata-rata | F-test | Sign |
| --- | --- | --- | --- | --- | --- | --- | --- |
| Y1.1 | 4 | 4.324324 | 4.375 | 4.5 | 4.299831 | 0.268 | 0.848 |
| Y1.2 | 4.5 | 4.486486 | 4.4375 | 4.5 | 4.480997 | 0.029 | 0.993 |
| Y1.3 | 4.5 | 4.216216 | 4.375 | 4.5 | 4.397804 | 0.411 | 0.746 |
| Y1.4 | 4.5 | 4.243243 | 4.4375 | 4.5 | 4.420186 | 0.637 | 0.595 |
| Y1.5 | 4.5 | 4.189189 | 4.375 | 4.5 | 4.391047 | 0.601 | 0.617 |
| Y1.6 | 4.5 | 4.216216 | 4.5 | 4.5 | 4.429054 | 0.794 | 0.503 |
| Y1.7 | 4.5 | 4.648649 | 4.5625 | 4.5 | 4.552787 | 0.141 | 0.935 |
| Y1.8 | 4.5 | 4.459459 | 4.5625 | 4.5 | 4.50549 | 0.104 | 0.957 |

| Usia | <21 tahun | 21-30 tahun | 31-40 tahun | 41-50 tahun | Rata-rata | F-test | Sign |
| --- | --- | --- | --- | --- | --- | --- | --- |
| Y2.1 | 4.5 | 4.513514 | 4.5625 | 4 | 4.394003 | 0.636 | 0.595 |
| Y2.2 | 4.5 | 4.540541 | 4.375 | 4.5 | 4.478885 | 0.34 | 0.797 |
| Y2.3 | 5 | 4.486486 | 4.625 | 4.5 | 4.652872 | 0.654 | 0.584 |
| Y2.4 | 5 | 4.513514 | 4.5625 | 4.5 | 4.644003 | 0.594 | 0.622 |
| Y2.5 | 5 | 4.324324 | 4.4375 | 4.5 | 4.565456 | 1.028 | 0.388 |
| Y2.6 | 5 | 4.432432 | 4.625 | 4.5 | 4.639358 | 0.932 | 0.432 |
| Y2.7 | 5 | 4.486486 | 4.375 | 4.5 | 4.590372 | 0.818 | 0.49 |
| Y2.8 | 4.5 | 4.486486 | 4.6875 | 4.5 | 4.543497 | 0.518 | 0.672 |
| Y2.9 | 5 | 4.486486 | 4.625 | 4.5 | 4.652872 | 0.738 | 0.534 |
| Y2.10 | 5 | 4.540541 | 4.6875 | 4.5 | 4.68201 | 0.692 | 0.561 |

| Jenis Kelamin | Laki-laki | Perempuan | Rata-rata | F-test | Sign |
| --- | --- | --- | --- | --- | --- |
| X1.1 | 4.571429 | 4.372093 | 4.471761 | 0.983 | 0.326 |
| X1.2 | 4.357143 | 4.325581 | 4.341362 | 0.02 | 0.888 |
| X1.3 | 4.428571 | 4.488372 | 4.458472 | 0.114 | 0.737 |
| X1.4 | 4.571429 | 4.27907 | 4.425249 | 1.904 | 0.173 |
| X1.5 | 4.285714 | 4.372093 | 4.328904 | 0.174 | 0.678 |
| X1.6 | 4.357143 | 4.418605 | 4.387874 | 0.14 | 0.71 |
| X1.7 | 4.428571 | 4.302326 | 4.365449 | 0.411 | 0.524 |
| X1.8 | 4.5 | 4.465116 | 4.482558 | 0.044 | 0.835 |
| X1.9 | 4.357143 | 4.395349 | 4.376246 | 0.055 | 0.816 |
| X1.10 | 4.428571 | 4.55814 | 4.493355 | 0.608 | 0.439 |
| X1.11 | 4.142857 | 4.232558 | 4.187708 | 0.184 | 0.67 |
| X1.12 | 4.285714 | 4.162791 | 4.224252 | 0.355 | 0.554 |
| X1.13 | 4.642857 | 4.534884 | 4.58887 | 0.426 | 0.517 |

| Jenis Kelamin | Laki-laki | Perempuan | Rata-rata | F-test | Sign |
| --- | --- | --- | --- | --- | --- |
| X2.1 | 4.571429 | 4.813953 | 4.692691 | 3.437 | 0.069 |
| X2.2 | 4.5 | 4.767442 | 4.633721 | 3.718 | 0.059 |
| X2.3 | 4.285714 | 4.55814 | 4.421927 | 2.215 | 0.142 |
| X2.4 | 4.285714 | 4.55814 | 4.421927 | 1.837 | 0.181 |
| X2.5 | 4.642857 | 4.767442 | 4.70515 | 0.7 | 0.407 |
| X2.6 | 4.714286 | 4.767442 | 4.740864 | 0.131 | 0.719 |
| X2.7 | 4.642857 | 4.767442 | 4.70515 | 0.7 | 0.407 |
| X2.8 | 4.357143 | 4.372093 | 4.364618 | 0.005 | 0.945 |
| X2.9 | 4.571429 | 4.604651 | 4.58804 | 0.036 | 0.85 |

| Jenis Kelamin | Laki-laki | Perempuan | Rata-rata | F-test | Sign |
| --- | --- | --- | --- | --- | --- |
| X3.1 | 4.428571 | 4.27907 | 4.353821 | 0.588 | 0.446 |
| X3.2 | 4.428571 | 4.488372 | 4.458472 | 0.114 | 0.737 |
| X3.3 | 4.285714 | 4.44186 | 4.363787 | 0.811 | 0.372 |
| X3.4 | 4.428571 | 4.674419 | 4.551495 | 2.081 | 0.155 |
| X3.5 | 4.142857 | 4.186047 | 4.164452 | 0.041 | 0.84 |
| X3.6 | 4.357143 | 4.511628 | 4.434385 | 0.772 | 0.383 |
| X3.7 | 4.357143 | 4.511628 | 4.434385 | 0.695 | 0.408 |
| X3.8 | 4.285714 | 4.44186 | 4.363787 | 0.66 | 0.42 |
| X3.9 | 4.357143 | 4.44186 | 4.399502 | 0.234 | 0.631 |
| X3.10 | 4.357143 | 4.372093 | 4.364618 | 0.007 | 0.935 |
| X3.11 | 4.428571 | 4.44186 | 4.435216 | 0.006 | 0.94 |
| X3.12 | 4.214286 | 4.372093 | 4.293189 | 0.548 | 0.462 |
| X3.13 | 4.285714 | 4.465116 | 4.375415 | 0.794 | 0.377 |

| Jenis Kelamin | Laki-laki | Perempuan | Rata-rata | F-test | Sign |
| --- | --- | --- | --- | --- | --- |
| Y1.1 | 4.285714 | 4.348837 | 4.317276 | 0.112 | 0.739 |
| Y1.2 | 4.428571 | 4.488372 | 4.458472 | 0.114 | 0.737 |
| Y1.3 | 4.285714 | 4.27907 | 4.282392 | 0.001 | 0.973 |
| Y1.4 | 4.071429 | 4.395349 | 4.233389 | 4.008 | 0.05 |
| Y1.5 | 4 | 4.348837 | 4.174419 | 3.978 | 0.051 |
| Y1.6 | 4 | 4.418605 | 4.209302 | 4.531 | **0.038** |
| Y1.7 | 4.5 | 4.651163 | 4.575581 | 0.769 | 0.384 |
| Y1.8 | 4.357143 | 4.534884 | 4.446013 | 0.922 | 0.341 |

| Jenis Kelamin | Laki-laki | Perempuan | Rata-rata | F-test | Sign |
| --- | --- | --- | --- | --- | --- |
| Y2.1 | 4.428571 | 4.534884 | 4.481728 | 0.407 | 0.526 |
| Y2.2 | 4.571429 | 4.465116 | 4.518272 | 0.407 | 0.526 |
| Y2.3 | 4.5 | 4.55814 | 4.52907 | 0.108 | 0.743 |
| Y2.4 | 4.5 | 4.55814 | 4.52907 | 0.139 | 0.711 |
| Y2.5 | 4.285714 | 4.418605 | 4.352159 | 0.592 | 0.445 |
| Y2.6 | 4.285714 | 4.581395 | 4.433555 | 2.932 | 0.092 |
| Y2.7 | 4.5 | 4.465116 | 4.482558 | 0.044 | 0.835 |
| Y2.8 | 4.357143 | 4.604651 | 4.480897 | 2.297 | 0.135 |
| Y2.9 | 4.428571 | 4.581395 | 4.504983 | 0.854 | 0.36 |
| Y2.10 | 4.5 | 4.627907 | 4.563953 | 0.611 | 0.438 |

| Masa Kerja | 1-5 tahun | 6-10 tahun | >10 tahun | Rata-rata | F-test | Sign |
| --- | --- | --- | --- | --- | --- | --- |
| X1.1 | 4.454545 | 4.4 | 4 | 4.284848 | 0.678 | 0.512 |
| X1.2 | 4.340909 | 4.4 | 4 | 4.24697 | 0.363 | 0.697 |
| X1.3 | 4.5 | 4.5 | 4 | 4.333333 | 1.096 | 0.341 |
| X1.4 | 4.340909 | 4.5 | 4 | 4.280303 | 0.61 | 0.547 |
| X1.5 | 4.318182 | 4.5 | 4.333333 | 4.383838 | 0.295 | 0.745 |
| X1.6 | 4.431818 | 4.4 | 4 | 4.277273 | 0.931 | 0.4 |
| X1.7 | 4.363636 | 4.3 | 4 | 4.221212 | 0.466 | 0.63 |
| X1.8 | 4.522727 | 4.4 | 4 | 4.307576 | 1.463 | 0.241 |
| X1.9 | 4.409091 | 4.4 | 4 | 4.269697 | 0.848 | 0.434 |
| X1.10 | 4.5 | 4.7 | 4.333333 | 4.511111 | 0.76 | 0.473 |
| X1.11 | 4.227273 | 4.2 | 4 | 4.142424 | 0.156 | 0.856 |
| X1.12 | 4.227273 | 4.2 | 3.666667 | 4.031313 | 0.994 | 0.377 |
| X1.13 | 4.590909 | 4.6 | 4 | 4.39697 | 1.793 | 0.176 |

| Masa Kerja | 1-5 tahun | 6-10 tahun | >10 tahun | Rata-rata | F-test | Sign |
| --- | --- | --- | --- | --- | --- | --- |
| X2.1 | 4.727273 | 4.8 | 5 | 4.842424 | 0.612 | 0.546 |
| X2.2 | 4.659091 | 4.8 | 5 | 4.819697 | 1.042 | 0.36 |
| X2.3 | 4.431818 | 4.7 | 4.666667 | 4.599495 | 0.943 | 0.396 |
| X2.4 | 4.409091 | 4.7 | 5 | 4.70303 | 1.792 | 0.176 |
| X2.5 | 4.681818 | 4.9 | 5 | 4.860606 | 1.317 | 0.276 |
| X2.6 | 4.727273 | 4.8 | 5 | 4.842424 | 0.513 | 0.602 |
| X2.7 | 4.681818 | 4.9 | 5 | 4.860606 | 1.317 | 0.276 |
| X2.8 | 4.409091 | 4.3 | 4 | 4.236364 | 0.532 | 0.59 |
| X2.9 | 4.545455 | 4.7 | 5 | 4.748485 | 1.127 | 0.331 |

| Masa Kerja | 1-5 tahun | 6-10 tahun | >10 tahun | Rata-rata | F-test | Sign |
| --- | --- | --- | --- | --- | --- | --- |
| X3.1 | 4.295455 | 4.5 | 4 | 4.265152 | 0.819 | 0.446 |
| X3.2 | 4.477273 | 4.6 | 4 | 4.359091 | 1.295 | 0.282 |
| X3.3 | 4.409091 | 4.4 | 4.333333 | 4.380808 | 0.025 | 0.976 |
| X3.4 | 4.568182 | 4.8 | 4.666667 | 4.678283 | 0.707 | 0.498 |
| X3.5 | 4.204545 | 4.1 | 4 | 4.101515 | 0.193 | 0.825 |
| X3.6 | 4.477273 | 4.6 | 4 | 4.359091 | 1.295 | 0.282 |
| X3.7 | 4.454545 | 4.6 | 4.333333 | 4.462626 | 0.317 | 0.729 |
| X3.8 | 4.386364 | 4.4 | 4.666667 | 4.484343 | 0.277 | 0.759 |
| X3.9 | 4.431818 | 4.5 | 4 | 4.310606 | 0.936 | 0.399 |
| X3.10 | 4.363636 | 4.5 | 4 | 4.287879 | 0.84 | 0.437 |
| X3.11 | 4.431818 | 4.6 | 4 | 4.343939 | 1.318 | 0.276 |
| X3.12 | 4.340909 | 4.4 | 4 | 4.24697 | 0.391 | 0.679 |
| X3.13 | 4.409091 | 4.5 | 4.333333 | 4.414141 | 0.104 | 0.901 |

| Masa Kerja | 1-5 tahun | 6-10 tahun | >10 tahun | Rata-rata | F-test | Sign |
| --- | --- | --- | --- | --- | --- | --- |
| Y1.1 | 4.363636 | 4.3 | 4 | 4.221212 | 0.512 | 0.602 |
| Y1.2 | 4.5 | 4.5 | 4 | 4.333333 | 1.096 | 0.341 |
| Y1.3 | 4.295455 | 4.2 | 4.333333 | 4.276263 | 0.105 | 0.901 |
| Y1.4 | 4.340909 | 4.3 | 4 | 4.213636 | 0.556 | 0.577 |
| Y1.5 | 4.25 | 4.4 | 4 | 4.216667 | 0.583 | 0.562 |
| Y1.6 | 4.272727 | 4.4 | 4.666667 | 4.446465 | 0.592 | 0.557 |
| Y1.7 | 4.613636 | 4.7 | 4.333333 | 4.54899 | 0.487 | 0.617 |
| Y1.8 | 4.454545 | 4.7 | 4.333333 | 4.49596 | 0.782 | 0.463 |

| Masa Kerja | 1-5 tahun | 6-10 tahun | >10 tahun | Rata-rata | F-test | Sign |
| --- | --- | --- | --- | --- | --- | --- |
| Y2.1 | 4.5 | 4.6 | 4.333333 | 4.477778 | 0.301 | 0.742 |
| Y2.2 | 4.522727 | 4.5 | 4 | 4.340909 | 1.34 | 0.27 |
| Y2.3 | 4.522727 | 4.7 | 4.333333 | 4.518687 | 0.603 | 0.551 |
| Y2.4 | 4.590909 | 4.5 | 4 | 4.363636 | 2.064 | 0.137 |
| Y2.5 | 4.409091 | 4.4 | 4 | 4.269697 | 0.749 | 0.478 |
| Y2.6 | 4.477273 | 4.7 | 4.333333 | 4.503535 | 0.763 | 0.471 |
| Y2.7 | 4.5 | 4.5 | 4 | 4.333333 | 1.238 | 0.298 |
| Y2.8 | 4.522727 | 4.7 | 4.333333 | 4.518687 | 0.68 | 0.511 |
| Y2.9 | 4.545455 | 4.6 | 4.333333 | 4.492929 | 0.278 | 0.758 |
| Y2.10 | 4.590909 | 4.7 | 4.333333 | 4.541414 | 0.554 | 0.578 |

| Tingkat Pendidikan | SMA/K Sederajat | D3 Sederajat | S1 Sederajat | S2 Sederajat | Rata-rata | F-test | Sign |
| --- | --- | --- | --- | --- | --- | --- | --- |
| X1.1 | 4.857143 | 5 | 4.333333 | 5 | 4.797619 | 1.946 | 0.133 |
| X1.2 | 4.714286 | 5 | 4.25 | 5 | 4.741071 | 1.496 | 0.226 |
| X1.3 | 4.714286 | 5 | 4.416667 | 5 | 4.782738 | 1.153 | 0.337 |
| X1.4 | 4.571429 | 5 | 4.291667 | 5 | 4.715774 | 0.932 | 0.432 |
| X1.5 | 4.571429 | 5 | 4.291667 | 5 | 4.715774 | 1.01 | 0.395 |
| X1.6 | 4.571429 | 5 | 4.375 | 4 | 4.486607 | 0.891 | 0.452 |
| X1.7 | 4.714286 | 5 | 4.270833 | 4 | 4.49628 | 1.486 | 0.229 |
| X1.8 | 4.857143 | 5 | 4.395833 | 5 | 4.813244 | 2.31 | 0.087 |
| X1.9 | 4.714286 | 5 | 4.333333 | 4 | 4.511905 | 1.772 | 0.164 |
| X1.10 | 4.714286 | 5 | 4.479167 | 5 | 4.798363 | 0.92 | 0.437 |
| X1.11 | 4.571429 | 5 | 4.145833 | 4 | 4.429315 | 1.327 | 0.275 |
| X1.12 | 4.571429 | 5 | 4.125 | 4 | 4.424107 | 1.472 | 0.233 |
| X1.13 | 4.857143 | 5 | 4.5 | 5 | 4.839286 | 1.401 | 0.253 |

| Tingkat Pendidikan | SMA/K Sederajat | D3 Sederajat | S1 Sederajat | S2 Sederajat | Rata-rata | F-test | Sign |
| --- | --- | --- | --- | --- | --- | --- | --- |
| X2.1 | 4.714286 | 5 | 4.75 | 5 | 4.866071 | 0.225 | 0.879 |
| X2.2 | 4.857143 | 5 | 4.666667 | 5 | 4.880952 | 0.622 | 0.604 |
| X2.3 | 4.428571 | 5 | 4.479167 | 5 | 4.726935 | 0.495 | 0.687 |
| X2.4 | 4.714286 | 5 | 4.4375 | 5 | 4.787946 | 0.764 | 0.52 |
| X2.5 | 4.857143 | 5 | 4.708333 | 5 | 4.891369 | 0.386 | 0.764 |
| X2.6 | 4.857143 | 5 | 4.729167 | 5 | 4.896577 | 0.322 | 0.809 |
| X2.7 | 4.857143 | 5 | 4.708333 | 5 | 4.891369 | 0.386 | 0.764 |
| X2.8 | 4.571429 | 5 | 4.333333 | 4 | 4.47619 | 0.591 | 0.624 |
| X2.9 | 4.571429 | 5 | 4.625 | 3 | 4.299107 | 3.253 | **0.029** |

| Tingkat Pendidikan | SMA/K Sederajat | D3 Sederajat | S1 Sederajat | S2 Sederajat | Rata-rata | F-test | Sign |
| --- | --- | --- | --- | --- | --- | --- | --- |
| X3.1 | 4.571429 | 5 | 4.25 | 5 | 4.705357 | 1.366 | 0.263 |
| X3.2 | 4.714286 | 5 | 4.416667 | 5 | 4.782738 | 1.153 | 0.337 |
| X3.3 | 4.714286 | 5 | 4.333333 | 5 | 4.761905 | 1.783 | 0.162 |
| X3.4 | 4.714286 | 5 | 4.583333 | 5 | 4.824405 | 0.427 | 0.734 |
| X3.5 | 4.428571 | 5 | 4.104167 | 5 | 4.633185 | 1.499 | 0.226 |
| X3.6 | 4.714286 | 5 | 4.416667 | 5 | 4.782738 | 1.153 | 0.337 |
| X3.7 | 4.714286 | 5 | 4.416667 | 5 | 4.782738 | 1.032 | 0.386 |
| X3.8 | 4.714286 | 5 | 4.333333 | 5 | 4.761905 | 1.428 | 0.245 |
| X3.9 | 4.714286 | 5 | 4.354167 | 5 | 4.767113 | 1.601 | 0.2 |
| X3.10 | 4.714286 | 5 | 4.3125 | 4 | 4.506696 | 1.516 | 0.221 |
| X3.11 | 4.714286 | 5 | 4.375 | 5 | 4.772321 | 1.437 | 0.242 |
| X3.12 | 4.714286 | 5 | 4.25 | 5 | 4.741071 | 1.619 | 0.196 |
| X3.13 | 4.571429 | 5 | 4.395833 | 4 | 4.491815 | 0.534 | 0.661 |

| Tingkat Pendidikan | SMA/K Sederajat | D3 Sederajat | S1 Sederajat | S2 Sederajat | Rata-rata | F-test | Sign |
| --- | --- | --- | --- | --- | --- | --- | --- |
| Y1.1 | 4.428571 | 5 | 4.291667 | 5 | 4.68006 | 0.932 | 0.432 |
| Y1.2 | 4.571429 | 5 | 4.4375 | 5 | 4.752232 | 0.689 | 0.563 |
| Y1.3 | 4.571429 | 5 | 4.229167 | 4 | 4.450149 | 1.151 | 0.337 |
| Y1.4 | 4.285714 | 5 | 4.291667 | 5 | 4.644345 | 1.117 | 0.35 |
| Y1.5 | 4.285714 | 5 | 4.25 | 4 | 4.383929 | 0.598 | 0.619 |
| Y1.6 | 4.428571 | 5 | 4.270833 | 5 | 4.674851 | 0.855 | 0.47 |
| Y1.7 | 4.714286 | 5 | 4.583333 | 5 | 4.824405 | 0.427 | 0.734 |
| Y1.8 | 4.714286 | 5 | 4.4375 | 5 | 4.787946 | 0.922 | 0.436 |

| Tingkat Pendidikan | SMA/K Sederajat | D3 Sederajat | S1 Sederajat | S2 Sederajat | Rata-rata | F-test | Sign |
| --- | --- | --- | --- | --- | --- | --- | --- |
| Y2.1 | 4.571429 | 5 | 4.479167 | 5 | 4.762649 | 0.622 | 0.604 |
| Y2.2 | 4.714286 | 5 | 4.4375 | 5 | 4.787946 | 1.164 | 0.332 |
| Y2.3 | 4.857143 | 5 | 4.479167 | 5 | 4.834077 | 1.368 | 0.262 |
| Y2.4 | 4.714286 | 5 | 4.5 | 5 | 4.803571 | 0.936 | 0.43 |
| Y2.5 | 4.714286 | 5 | 4.3125 | 5 | 4.756696 | 1.984 | 0.128 |
| Y2.6 | 4.714286 | 5 | 4.458333 | 5 | 4.793155 | 0.917 | 0.439 |
| Y2.7 | 4.714286 | 5 | 4.416667 | 5 | 4.782738 | 1.305 | 0.282 |
| Y2.8 | 4.571429 | 5 | 4.520833 | 5 | 4.773065 | 0.503 | 0.682 |
| Y2.9 | 4.714286 | 5 | 4.5 | 5 | 4.803571 | 0.815 | 0.491 |
| Y2.10 | 4.857143 | 5 | 4.541667 | 5 | 4.849702 | 1.131 | 0.345 |

| Unit Kerja | PAUD | SD | SMP-SMK | Rata-rata | F-test | Sign |
| --- | --- | --- | --- | --- | --- | --- |
| X1.1 | 4.75 | 4.24 | 4.5 | 4.496667 | 2.246 | 0.116 |
| X1.2 | 4.75 | 4.16 | 4.375 | 4.428333 | 2.224 | 0.118 |
| X1.3 | 4.75 | 4.36 | 4.5 | 4.536667 | 1.487 | 0.235 |
| X1.4 | 4.5 | 4.2 | 4.458333 | 4.386111 | 1.065 | 0.352 |
| X1.5 | 4.625 | 4.2 | 4.416667 | 4.413889 | 1.451 | 0.243 |
| X1.6 | 4.625 | 4.32 | 4.416667 | 4.453889 | 1.018 | 0.368 |
| X1.7 | 4.75 | 4.16 | 4.375 | 4.428333 | 2.876 | 0.065 |
| X1.8 | 4.75 | 4.28 | 4.583333 | 4.537778 | 3.451 | 0.039 |
| X1.9 | 4.625 | 4.24 | 4.458333 | 4.441111 | 2.092 | 0.133 |
| X1.10 | 4.625 | 4.52 | 4.5 | 4.548333 | 0.16 | 0.853 |
| X1.11 | 4.75 | 4 | 4.25 | 4.333333 | 4.263 | 0.019 |
| X1.12 | 4.875 | 3.96 | 4.208333 | 4.347778 | 6.935 | 0.002 |
| X1.13 | 4.875 | 4.4 | 4.625 | 4.633333 | 2.858 | 0.066 |

| Unit Kerja | PAUD | SD | SMP-SMK | Rata-rata | F-test | Sign |
| --- | --- | --- | --- | --- | --- | --- |
| X2.1 | 5 | 4.72 | 4.708333 | 4.809444 | 1.521 | 0.228 |
| X2.2 | 5 | 4.72 | 4.583333 | 4.767778 | 2.623 | 0.082 |
| X2.3 | 4.75 | 4.4 | 4.5 | 4.55 | 1.032 | 0.363 |
| X2.4 | 4.75 | 4.52 | 4.375 | 4.548333 | 1.018 | 0.368 |
| X2.5 | 4.875 | 4.76 | 4.666667 | 4.767222 | 0.601 | 0.552 |
| X2.6 | 4.875 | 4.76 | 4.708333 | 4.781111 | 0.366 | 0.695 |
| X2.7 | 5 | 4.76 | 4.625 | 4.795 | 1.923 | 0.156 |
| X2.8 | 4.625 | 4.24 | 4.416667 | 4.427222 | 1.023 | 0.367 |
| X2.9 | 4.75 | 4.64 | 4.5 | 4.63 | 0.718 | 0.492 |

| Unit Kerja | PAUD | SD | SMP-SMK | Rata-rata | F-test | Sign |
| --- | --- | --- | --- | --- | --- | --- |
| X3.1 | 4.5 | 4.32 | 4.25 | 4.356667 | 0.462 | 0.632 |
| X3.2 | 4.625 | 4.48 | 4.416667 | 4.507222 | 0.394 | 0.676 |
| X3.3 | 4.625 | 4.4 | 4.333333 | 4.452778 | 0.802 | 0.454 |
| X3.4 | 4.625 | 4.72 | 4.5 | 4.615 | 0.948 | 0.394 |
| X3.5 | 4.375 | 4 | 4.291667 | 4.222222 | 1.535 | 0.225 |
| X3.6 | 4.625 | 4.44 | 4.458333 | 4.507778 | 0.326 | 0.723 |
| X3.7 | 4.75 | 4.36 | 4.5 | 4.536667 | 1.333 | 0.272 |
| X3.8 | 4.625 | 4.32 | 4.416667 | 4.453889 | 0.729 | 0.487 |
| X3.9 | 4.625 | 4.32 | 4.458333 | 4.467778 | 0.971 | 0.385 |
| X3.10 | 4.625 | 4.32 | 4.333333 | 4.426111 | 0.89 | 0.417 |
| X3.11 | 4.625 | 4.44 | 4.375 | 4.48 | 0.573 | 0.567 |
| X3.12 | 4.5 | 4.36 | 4.25 | 4.37 | 0.418 | 0.66 |
| X3.13 | 4.625 | 4.4 | 4.375 | 4.466667 | 0.454 | 0.638 |

| Unit Kerja | PAUD | SD | SMP-SMK | Rata-rata | F-test | Sign |
| --- | --- | --- | --- | --- | --- | --- |
| Y1.1 | 4.5 | 4.2 | 4.416667 | 4.372222 | 1.134 | 0.329 |
| Y1.2 | 4.5 | 4.4 | 4.541667 | 4.480556 | 0.379 | 0.686 |
| Y1.3 | 4.75 | 4.04 | 4.375 | 4.388333 | 5.112 | **0.009** |
| Y1.4 | 4.625 | 4.2 | 4.333333 | 4.386111 | 1.966 | 0.15 |
| Y1.5 | 4.5 | 4.16 | 4.291667 | 4.317222 | 1.082 | 0.346 |
| Y1.6 | 4.75 | 4.28 | 4.208333 | 4.412778 | 2.181 | 0.123 |
| Y1.7 | 4.75 | 4.6 | 4.583333 | 4.644444 | 0.273 | 0.762 |
| Y1.8 | 4.625 | 4.48 | 4.458333 | 4.521111 | 0.232 | 0.794 |

| Unit Kerja | PAUD | SD | SMP-SMK | Rata-rata | F-test | Sign |
| --- | --- | --- | --- | --- | --- | --- |
| Y2.1 | 4.375 | 4.52 | 4.541667 | 4.478889 | 0.289 | 0.75 |
| Y2.2 | 4.625 | 4.32 | 4.625 | 4.523333 | 2.36 | 0.104 |
| Y2.3 | 4.625 | 4.52 | 4.541667 | 4.562222 | 0.1 | 0.905 |
| Y2.4 | 4.625 | 4.48 | 4.583333 | 4.562778 | 0.372 | 0.691 |
| Y2.5 | 4.625 | 4.24 | 4.458333 | 4.441111 | 1.837 | 0.169 |
| Y2.6 | 4.625 | 4.48 | 4.5 | 4.535 | 0.195 | 0.824 |
| Y2.7 | 4.5 | 4.4 | 4.541667 | 4.480556 | 0.427 | 0.655 |
| Y2.8 | 4.625 | 4.6 | 4.458333 | 4.561111 | 0.524 | 0.595 |
| Y2.9 | 4.625 | 4.56 | 4.5 | 4.561667 | 0.177 | 0.838 |
| Y2.10 | 4.75 | 4.64 | 4.5 | 4.63 | 0.813 | 0.449 |
