# Supplementary figures and images for: Exploring Prajnamitra Maitreya Buddhists School Pekanbaru: Do leadership, work environment, and organisational culture influence the teachers’ competence and work performance?
Source: PLoS One. 2023 May 16;18(5):e0282941. doi: 10.1371/journal.pone.0282941 (PMC10187918; doi:10.1371/journal.pone.0282941)

X1
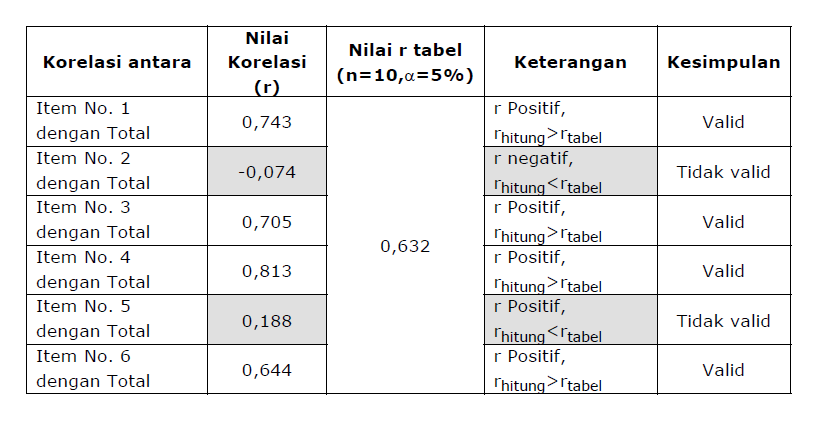


|  |  | n-13, alpa 5% |  |  |
| --- | --- | --- | --- | --- |
|  | 0.85099 | 0,553 |  |  |
|  | 0.881863 |  |  |  |
|  | 0.828744 |  |  |  |
|  | 0.777145 |  |  |  |
|  | 0.853295 |  |  |  |
|  | 0.714518 |  |  |  |
|  | 0.856697 |  |  |  |
|  | 0.816569 |  |  |  |
|  | 0.798437 |  |  |  |
|  | 0.764788 |  |  |  |
|  | 0.829168 |  |  |  |
|  | 0.743488 |  |  |  |
|  | 0.794674 |  |  |  |

X2

|  |  | n-13, alpa 5% |  |  |
| --- | --- | --- | --- | --- |
|  | 0.85099 | 0,668 |  |  |
|  | 0.881863 |  |  |  |
|  | 0.828744 |  |  |  |
|  | 0.777145 |  |  |  |
|  | 0.853295 |  |  |  |
|  | 0.714518 |  |  |  |
|  | 0.856697 |  |  |  |
|  | 0.816569 |  |  |  |
|  | 0.798437 |  |  |  |

X3

|  |  | n-13, alpa 5% |  |  |
| --- | --- | --- | --- | --- |
|  | 0.615229 | 0,553 |  |  |
|  | 0.711595 |  |  |  |
|  | 0.818248 |  |  |  |
|  | 0.768577 |  |  |  |
|  | 0.79257 |  |  |  |
|  | 0.83063 |  |  |  |
|  | 0.802584 |  |  |  |
|  | 0.848065 |  |  |  |
|  | 0.867684 |  |  |  |
|  | 0.869799 |  |  |  |
|  | 0.847775 |  |  |  |
|  | 0.848415 |  |  |  |
|  | 0.820161 |  |  |  |

Y1

|  |  | n-13, alpa 5% |  |  |
| --- | --- | --- | --- | --- |
|  | 0.804679 | 0,707 |  |  |
|  | 0.817843 |  |  |  |
|  | 0.652057 |  |  |  |
|  | 0.844464 |  |  |  |
|  | 0.840944 |  |  |  |
|  | 0.785978 |  |  |  |
|  | 0.81099 |  |  |  |
|  | 0.767008 |  |  |  |

Y2

|  |  | n-13, alpa 5% |  |  |
| --- | --- | --- | --- | --- |
|  | 0.766997 | 0,632 |  |  |
|  | 0.789313 |  |  |  |
|  | 0.905526 |  |  |  |
|  | 0.873294 |  |  |  |
|  | 0.841371 |  |  |  |
|  | 0.824324 |  |  |  |
|  | 0.890763 |  |  |  |
|  | 0.917213 |  |  |  |
|  | 0.931472 |  |  |  |
|  | 0.868579 |  |  |  |

Supplement: S2 File — (ZIP) [file pone.0282941.s002.zip › valid reliab.docx]

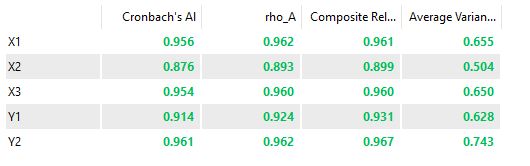


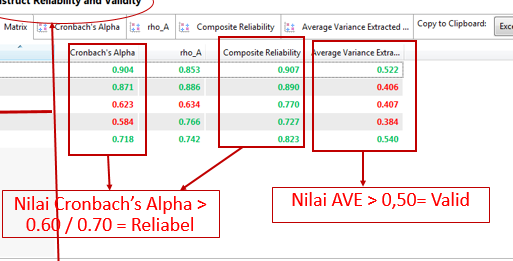


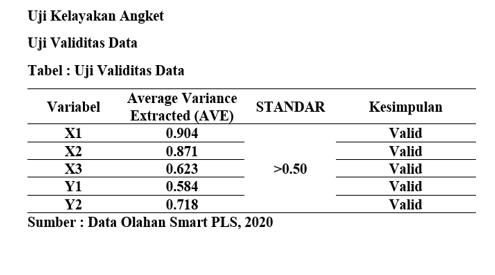


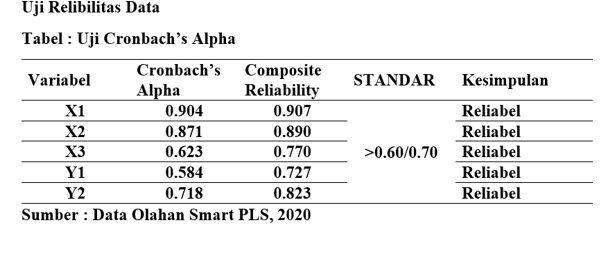


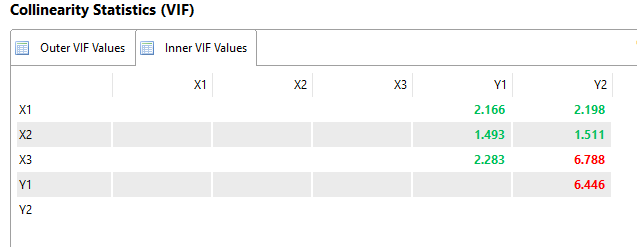


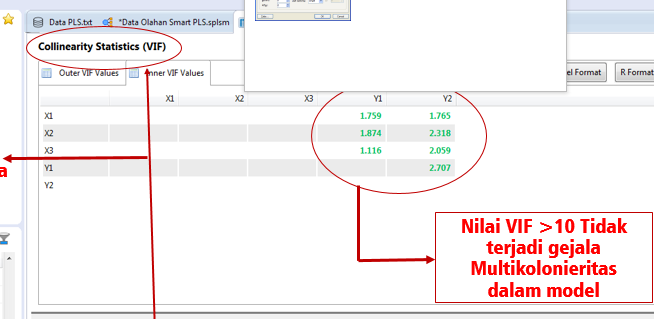


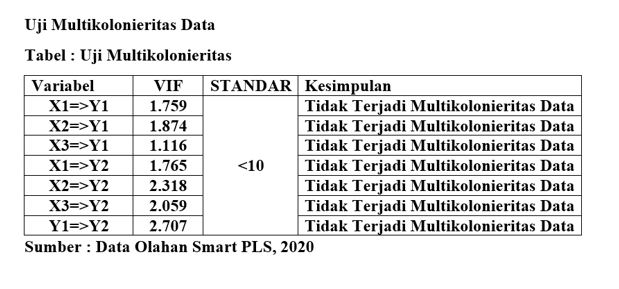


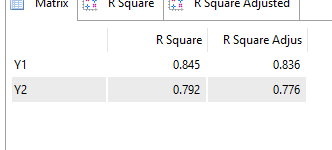


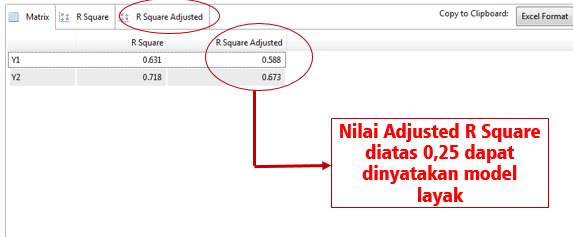


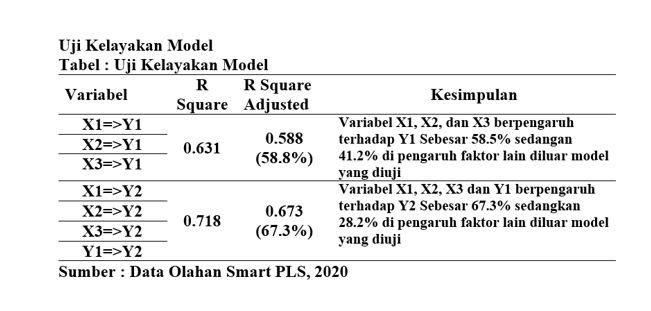


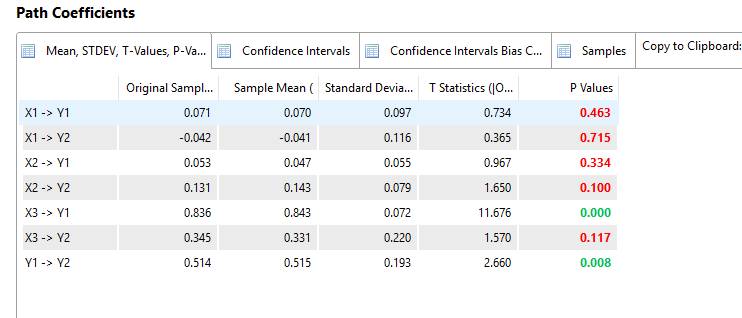


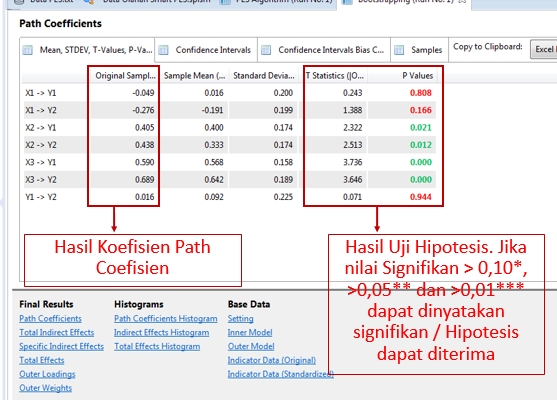


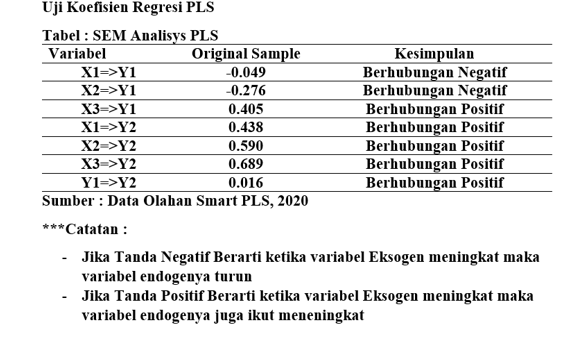


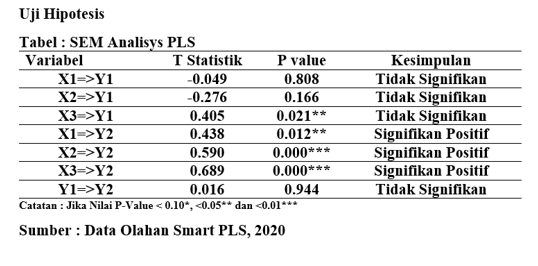
+++

Supplement: S2 File — (ZIP) [file pone.0282941.s002.zip › tes ulang/Tes Ulang PLs.docx]

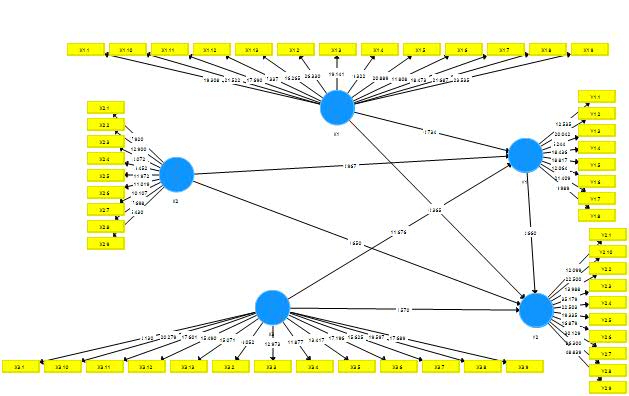

Supplement: S2 File — (ZIP) [file pone.0282941.s002.zip › tes ulang/TEs ulang.jpg]

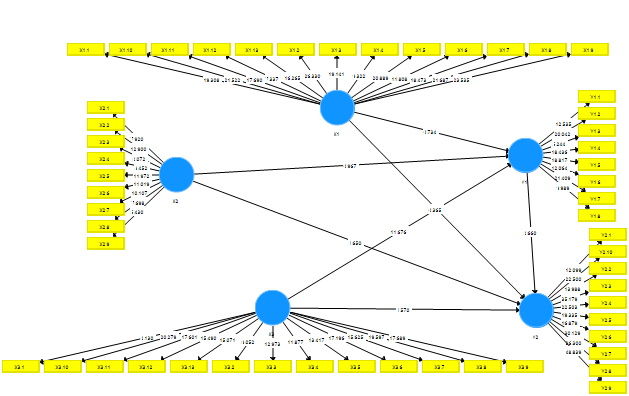

Supplement: S2 File — (ZIP) [file pone.0282941.s002.zip › tes ulang/Tes ulang.png]
